# A genomic hotspot of diversifying selection and structural change in the hoary bat (*Lasiurus cinereus*)

**DOI:** 10.7717/peerj.17482

**Published:** 2024-05-31

**Authors:** Robert S. Cornman

**Affiliations:** U.S. Geological Survey, Fort Collins Science Center, Fort Collins, Colorado, United States

**Keywords:** Hoary bat, Evolutionary rate analysis, Comparative genomics, Adaptation

## Abstract

**Background:**

Previous work found that numerous genes positively selected within the hoary bat (*Lasiurus cinereus*) lineage are physically clustered in regions of conserved synteny. Here I further validate and expand on those finding utilizing an updated *L. cinereus* genome assembly and additional bat species as well as other tetrapod outgroups.

**Methods:**

A chromosome-level assembly was generated by chromatin-contact mapping and made available by DNAZoo (www.dnazoo.org). The genomic organization of orthologous genes was extracted from annotation data for multiple additional bat species as well as other tetrapod clades for which chromosome-level assemblies were available from the National Center for Biotechnology Information (NCBI). Tests of branch-specific positive selection were performed for *L. cinereus* using PAML as well as with the HyPhy package for comparison.

**Results:**

Twelve genes exhibiting significant diversifying selection in the *L. cinereus* lineage were clustered within a 12-Mb genomic window; one of these (*Trpc4*) also exhibited diversifying selection in bats generally. Ten of the 12 genes are landmarks of two distinct blocks of ancient synteny that are not linked in other tetrapod clades. Bats are further distinguished by frequent structural rearrangements within these synteny blocks, which are rarely observed in other Tetrapoda. Patterns of gene order and orientation among bat taxa are incompatible with phylogeny as presently understood, implying parallel evolution or subsequent reversals. Inferences of positive selection were found to be robust to alternative phylogenetic topologies as well as a strong shift in background nucleotide composition in some taxa.

**Discussion:**

This study confirms and further localizes a genomic hotspot of protein-coding divergence in the hoary bat, one that also exhibits an increased tempo of structural change in bats compared with other mammals. Most genes in the two synteny blocks have elevated expression in brain tissue in humans and model organisms, and genetic studies implicate the selected genes in cranial and neurological development, among other functions.

## Introduction

As high-quality genome references with well-supported annotations continue to be released, it has become increasingly common to compare the evolution of genomic features within a clade, often in conjunction with ecological, life-history, or morphological data (Lewin et al., 2018). These comparative analyses can illuminate the genomic architecture of trait evolution, bringing to bear diverse data types such as cytogenetics, gene family evolution, evolutionary rates of orthologous genes, gene expression patterns, and methylation profiles (Wei et al., 2002). Protein evolutionary rates are among the most accessible and widely applied comparisons, and several tests have been proposed to identify positive diversifying selection within a gene tree (Muse & Gaut, 1994; Yang & Nielsen, 2000; Seo, Kishino & Thorne, 2004; Smith et al., 2015). As nonsynonymous substitution rates statistically higher than synonymous rates are only expected under adaptive evolution, ratio-based tests can confirm hypotheses of diversifying selection on candidate genes relevant to fitness (Aguileta et al., 2009) as well as discover episodes of selection agnostically by scanning whole genomes (Heger & Ponting, 2007; Kosiol et al., 2008). In addition to revealing mechanisms of adaptation, genomic surveys of substitution patterns can be important in other contexts such as evolutionary medicine, *e.g*. by quantifying levels of protein constraint (Lindblad-Toh et al., 2011) and estimating the functional significance of mutations (Cingolani et al., 2012).

A number of studies have investigated rates of protein evolution within the various orders of mammals, some of which (Shen et al., 2010; Hawkins et al., 2019; Cornman & Cryan, 2022, but see Jebb et al., 2020) have indicated high rates of positive selection on coding sequence in bats (order Chiroptera). A recent study (Cornman & Cryan, 2022) identified candidates of positive selection in the hoary bat lineage, including genes clustered in regions of conserved synteny (discrete regions in which ancestral gene sets are colinear in comparative genomic alignments despite extensive background divergence in genome content and organization). For example, six genes showed evidence of positive selection in the vicinity of the cat-eye critical region, which overlaps a conserved synteny block in tetrapods, so-named for its association with the ‘cat-eye’ spectrum of congenital developmental disorders in human (Footz et al., 2001). In addition to containing clustered signatures of positive selection, this region was structurally divergent in the hoary bat lineage as well, including rearrangements not apparent in other mammalian clades (Cornman & Cryan, 2022). Furthermore, several other genes associated with cranial dysmorphy in humans were selection candidates in *L. cinereus*, collectively indicating that cranial development may have been a phenotypic target of selection in the divergence of the hoary bat lineage (Cornman & Cryan, 2022). This hypothesis is consistent with previous morphometric studies that have documented associations between cranial morphology and ecological divergence among related bat species and at higher taxonomic levels in bats (Evin et al., 2008; Hedrick & Dumont, 2018; Arbour, Curtis & Santana, 2021).

While the hoary bat assembly analyzed by Cornman & Cryan (2022) had a high scaffold N50 (35.1 Mb; Cornman et al., 2021), contiguity was not at chromosomal level, such that clustering of selection candidates and conservation of synteny could not be fully evaluated. Few other chromosome-scale assemblies of bat genomes were available at that time, preventing a reconstruction of the sequence of structural changes. However, a recent scaffolding effort with “Hi-C” chromatin mapping (Belton et al., 2012) has revealed that two of the three clusters discussed by Cornman & Cryan (2022) form a contiguous block on a large metacentric chromosome. Additional high-quality genomes of bat species have also become available since that study, which enable improved sampling of gene trees and thus increased statistical power of selection tests within bats, as well as improved comparisons of synteny. Here I report that expanded ortholog alignments strengthen inferences of diversifying selection within the hoary bat lineage (*i.e*., since the divergence of genus *Lasiurus* from other analyzed genera). Co-localization of the two synteny blocks was not observed in a survey of other tetrapod orders and appears not to be the ancestral state of Chiroptera, yet has an apparently homoplasious distribution within this group, implying parallel evolution of complex features or a similarly complex reversal. Numerous smaller changes in gene order are found within these synteny blocks as well, particularly within suborder Yangochiroptera.

## Materials and Methods

The analysis of Cornman & Cryan (2022) was based on a 10X linked-read assembly (accession GCA_011751065.1 of the National Center for Biotechnology Information (NCBI), Cornman et al., 2021). This was subsequently scaffolded by the DNAZoo Consortium (Dudchenko et al., 2017) using a HiC chromatin contact-mapping data set (NCBI accession SRX8933264) and made publicly available at https://www.dnazoo.org/assemblies/Aeorestes_cinereus. (Note that *Aeroestes cinereus* and *Lasiurus cinereus* are synonymous; we follow the latter usage here as it predominates in the literature.) To evaluate assembly coverage patterns, confirm the X chromosome, and detect assembly artifacts such as collapsed repeats, an existing genomic short-read data set for *L. cinereus* (PRJNA559902; Pinzari et al., 2020) was aligned to the genome assembly with bowtie2 v. 2.4.5 (Langmead & Salzberg, 2012) using the “fast” and “end-to-end” parameter switches, then filtered with SAMtools v. 1.12 (Li et al., 2009) at a mapping quality of 30 (Phred-scaled). Mapped reads were summed per major scaffold using the bedcov function of SAMtools, then divided by chromosome length as well as the total number of reads mapped per sample to those scaffolds. The resulting values were then normalized to the average of all sample-scaffold pairs.

Coding-sequence alignments were generated from precomputed orthologs sets for each gene, available from NCBI *via* the corresponding gene pages, with the exception that *L. cinereus* orthologs were taken from Cornman & Cryan (2022). Some transcripts in the pre-computed ortholog groups differed structurally from the others due to alternative splicing, in which case substituting a different isoform of the same gene often sufficed to correct the alignment. In other cases, un-annotated exons could be extracted by searching genomic sequence with coding sequence of a related taxon. Rarely, the coding sequence of a congener was used when available and substantially more complete. If none of these alternatives yielded at least a partial coding sequence for a genus, that genus was deleted from the guide tree and evolutionary rates were computed from the remaining taxa. If multiple taxa were unavailable, the gene was not analyzed. Ortholog sets were aligned at the nucleotide level with MAFFT v. 7.480 (Katoh et al., 2002), trimmed of untranslated regions, and realigned at the protein level. Low-complexity or gapped regions for which codon-level orthology was questionable were deleted from alignments between unambiguously conserved codons. Analyzed ortholog alignments are available in File S1.

The guide tree for all evolutionary rate analyses reported in the Results follows the phylogenetic analysis of Amador et al. (2018), however the consistency of results was qualitatively assessed by examining three other tree topologies as well (Fig. S1). For example, Agnarsson et al. (2011) supported a closer relationship of *Lasiurus* and *Pipistrellus*, with *Eptesicus* an outgroup to the pair, which is also consistent with overall karyotype (Bickham, 1987). This alternative topology is labeled “tree 2” in Fig. S1. A third topology was tested in which the location of *Miniopterus* follows Agnarsson et al. (2011), as sister to phyllostomids rather than vespertilionids (“tree 3”). A fourth topology (“tree 4”) is a pruned version of tree 1, in which taxa with greatly increased GC content of some tested genes (see Results) were removed to ensure that this shift in nucleotide compositions did not influence the conclusions drawn.

Parameters and likelihoods of two models were estimated with PAML (Yang, 2007). Setting the control variables “Model = 0” and “NSsites = 0” estimates one rate class ω across the phylogeny, whereas setting the control variables “Model = 2” and “NSsites = 0” and labeling the *L. cinereus* branch as foreground estimates two ω values, one for background taxa and one for the foreground taxon. The test statistic for branch-specific positive selection was then calculated as twice the difference in log-likelihood between the latter and the former, assuming a χ^2^ distribution with one degree of freedom (Yang, 2007). False discovery rate (FDR) correction was performed with the Benjamini-Hochberg procedure of the R function p.adjust (R Core Team R, 2018). The aBSREL and BUSTED programs of the HyPhy package (Kosakovsky Pond et al., 2020) were also used to test for positive selection in order to evaluate overall consistency of inferences. False discovery was performed for the set of 34 tested genes (see Results), but separately for each tree topology and for each statistical test. This is because only the PAML results for tree 1 are reported in the Results; the other methods and topologies are reported for qualitative comparison only. FDR correction of aBSREL *P*-values were performed on the uncorrected values for the foreground branch only (*L. cinereus*). Model outputs are summarized in File S2.

Ortholog locations were extracted for each gene from the gene feature files accompanying each annotated genome downloaded from NCBI. Locations of *L. cinereus* genes were updated by splice-aware alignment of transcripts to the revised genome assembly with GMAP v. 2023-12-01 using default settings (Wu & Watanabe, 2005). Clustering of the original selection candidates from Cornman & Cryan (2022) was re-evaluated for the updated assembly in two ways. First, the cumulative proportions of selected and total genes were plotted in consecutive 1-Mb windows based on midpoint coordinate. Secondly, a null distribution for the expected number of selected genes in comparable windows was generated by selecting 1,000 random 12-Mb windows that were permitted to cross chromosome boundaries, with chromosomes concatenated in a random order for each iteration.

Bat “RefSeq” genome accessions generated by NCBI that were used in evolutionary rate and synteny analyses include *Eptesicus fuscus* (GCA_027574615.1; Paulat et al., 2023), *Pipistrellus kuhlii* (GCF_014108245.1; Jebb et al., 2020), *Myotis myotis* (GCF_014108235.1; Jebb et al., 2020), *Molossus molossus* (GCF_014108415.1; Jebb et al., 2020), *Artibeus jamaicensis* (GCF_021234435.1; Wang et al., 2020), *Phyllostomus discolor* (GCF_004126475.2; Jebb et al., 2020), *Rousettus aegyptiacus* (GCF_014176215.1; Jebb et al., 2020), and *Rhinolophus ferrumequinum* (GCF_004115265.2; Jebb et al., 2020). Bat genomes used for evolutionary rate analysis only include *Sturnira hondurensis* (GCF_014824575.3; Wang et al., 2020), *Pteropus alecto* (GCF_000325575.1; Zhang et al., 2013), *Miniopterus natalensis* (GCF_001595765.1; Eckalbar et al., 2016), and *Hipposideros armiger* (GCF_001890085.2; Dong et al., 2016). Genome accessions of outgroup tetrapods used in synteny analyses included *Homo sapiens* (GCF_000001405.40; International Human Genome Sequencing Consortium, 2001), *Mus musculus* (GCF_000001635.27; Church et al., 2009), *Corvus cornix* (GCF_000738735.6; Poelstra et al., 2014), *Monodelphis domestica* (GCF_027887165.1; Mikkelsen et al., 2007), *Ornithorhynchus anatinus* (GCF_004115215.2; Zhou et al., 2021), *Ochotona princeps* (GCF_030435755.1; Sjodin et al., 2021), *Sus scrofa* (GCF_000003025.6; Warr et al., 2020), *Bos taurus* (GCF_002263795.3; Rosen et al., 2020), *Equus caballus* (GCF_002863925.1; Kalbfleisch et al., 2018), and *Felis catus* (GCF_018350175.1; Pontius et al., 2007). Syntenic regions were evaluated using pre-computed data tracks of the University of California, Santa Cruz (UCSC) Genome Browser (Lee et al., 2020), including Gencode (Frankish et al., 2021) and RefSeq (O’Leary et al., 2016) annotation data for human.

Sequence analysis was performed to investigate the evolution of a set of genes with partial homology to the transcription factor *Tbx1* (see Results). Manipulation and visualization of these sequence alignments was performed with BioEdit (Hall, 1999), whereas protein secondary structure was predicted with Jpred4 (Drozdetskiy et al., 2015) and core promoter sequences identified with the neural network tool of Reese (2001).

Gene expression data were tabulated from two sources. Summaries of tissue-level expression in human and mouse orthologs were extracted from their NCBI Gene pages and are derived from Fagerberg et al. (2014) and Yue et al. (2014), respectively. Tissues of elevated expression for each gene were obtained from the “UP_Table” expression output of the DAVID functional annotation tool. Gene ontology enrichment was performed with the AmiGO 2 web service (Ashburner et al., 2000; The Gene Ontology Consortium et al., 2023) using official gene symbols as input and the GO-Slim ontology terms for biological process.

Primary sequence data underlying these analyses are also available in a U.S. Geological Survey data release (Cornman, 2024).

## Results and discussion

The updated *L. cinereus* genome assembly is comprised of 14 major scaffolds totaling 2.08 Gb, encompassing 98.9% of the total assembly length of 2.11 Gb. The assembly is similar in length to the chromosome-level assembly of the vespertilionid species *E. fuscus* (2.01 Gb) and matches the *L. cinereus* karyotype (Bickham, 1987) in terms of chromosome number and their relative lengths (Fig. S2). Among-individual coverage patterns in a population sample were consistent for all major scaffolds except Scaffold 10, which exhibited a bimodal coverage pattern unambiguously indicating the X chromosome. No scaffold was identified with coverage patterns indicative of the Y chromosome, as expected given that the sequenced individual was female. Although not relevant to the present study, the smallest scaffolds were consistently undersampled in the Illumina short-read libraries used for coverage assessment (Fig. S2), an unanticipated effect which could potentially bias population-genomic analyses in this or related species.

**Figure 1:**
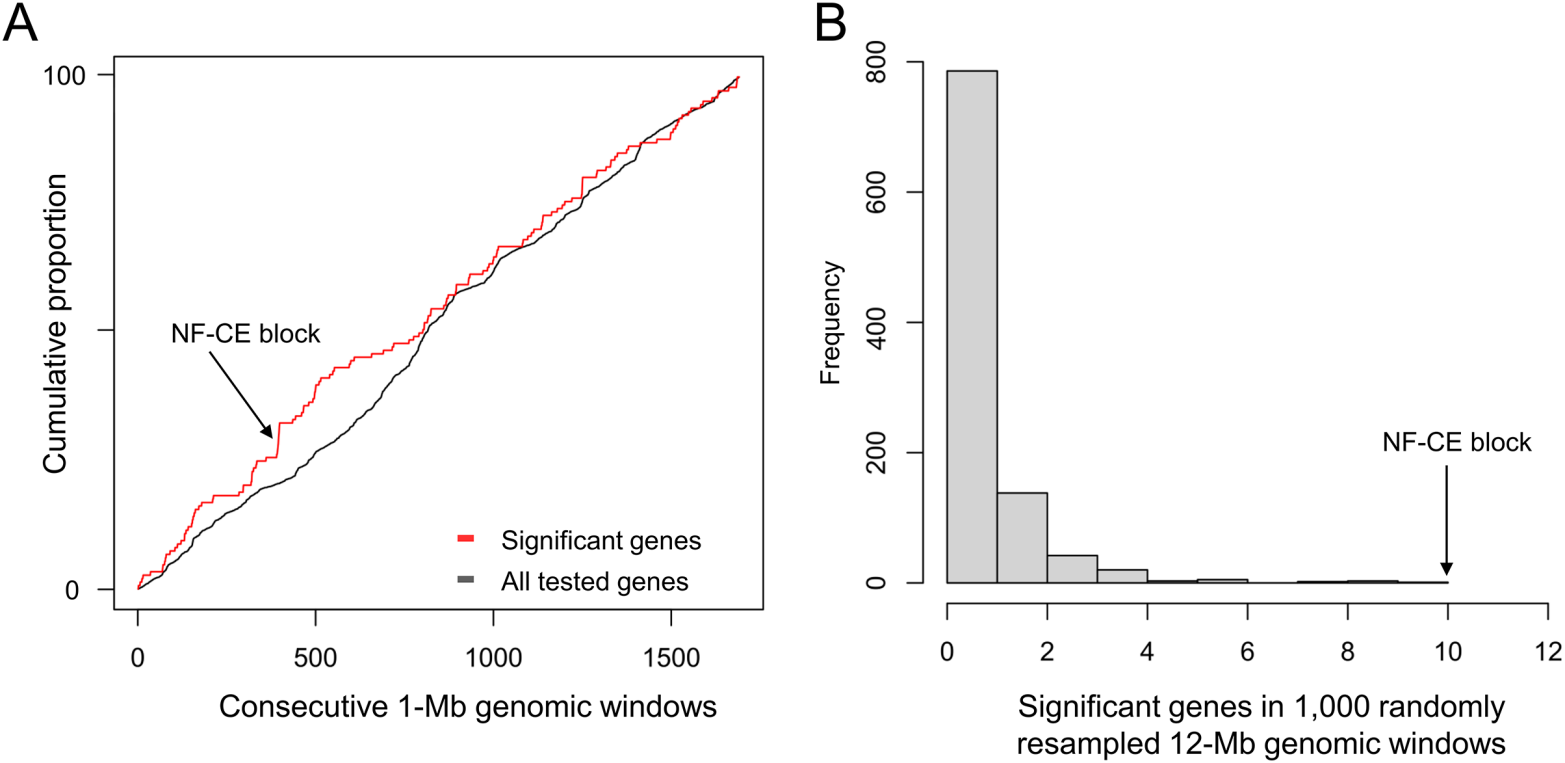
A pronounced cluster of positively selected genes occurs in the chromosome-level assembly of hoary bat (*Lasiurus cinereus*). The genomic region containing the cluster of selection candidates is labeled “NF-CE block”, see text for details. (A) Cumulative proportion of all single-copy orthologs tested for positive selection in a previous study (see text for details) compared with the cumulative proportion of genes with significant test results. (B) Histogram of the number of positive selection candidates in windows of comparable size to the 12-Mb region identified in panel A. The observed value for the NF-CE block is ten.

To re-evaluate the clustering of positive selection candidates in this new assembly, the 9,447 tested single-copy orthologs from Cornman & Cryan (2022) were re-aligned, with all but two placed on one of the 14 major scaffolds. The cumulative distribution of positive selection candidates diverges from all tested genes (Fig. 1A), particularly in the region of the synteny blocks described below, which encompasses ten such genes within a 12-Mb span. No randomly resampled 12-Mb windows selected from randomly concatenated chromosomes contained more than ten positive selection candidates, and 99.9% of resampled windows were less than this value (Fig. 1B).

**Figure 2:**
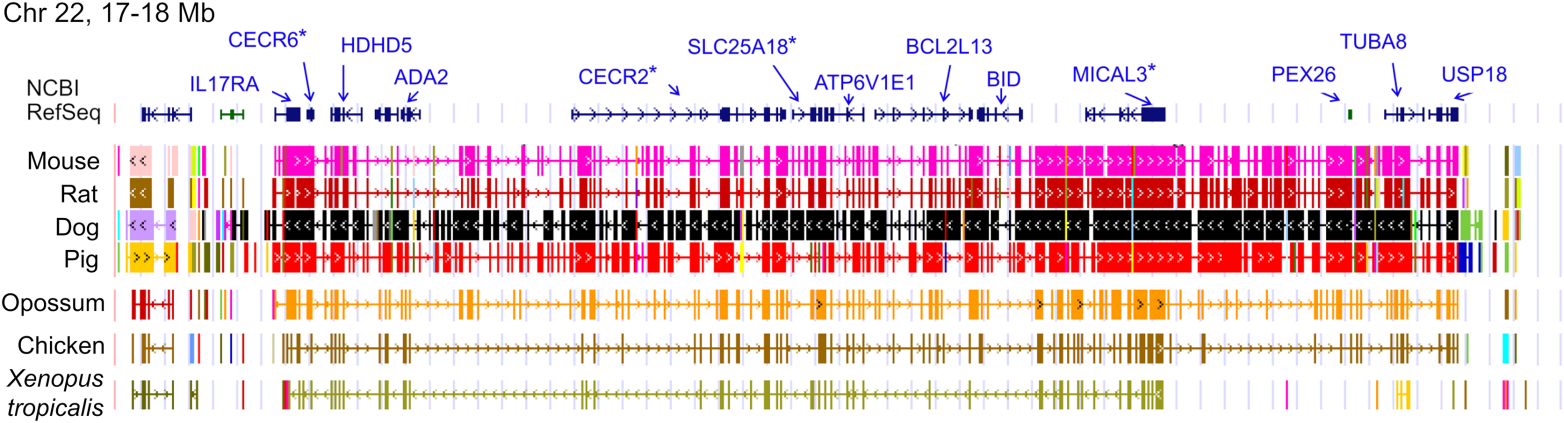
Demarcation of the cat-eye (CE) synteny block based on the gene order in human. Each row is a genomic data track derived from the University of California Santa Cruz (UCSC) Genome Browser, with the approximate location in the human genome indicated in the upper left. The top track shows ideograms of the human RefSeq genes curated by the National Center for Biotechnology Information (NCBI), indicating exon structure and orientation, labeled with the gene symbols used in the text. Asterisks indicate genes with evidence of positive selection within the *Lasiurus cinereus* branch of the tested phylogeny. The subsequent tracks identify blocks of conserved sequence in representative Tetrapoda of increasing evolutionary distance to human. Within each species, alignments on the same chromosome share a common color and are linked by flow lines if contiguous. Regions that are approximately uniform in color and contiguous within each species are syntenic. See Methods for data track sources.

**Figure 3:**
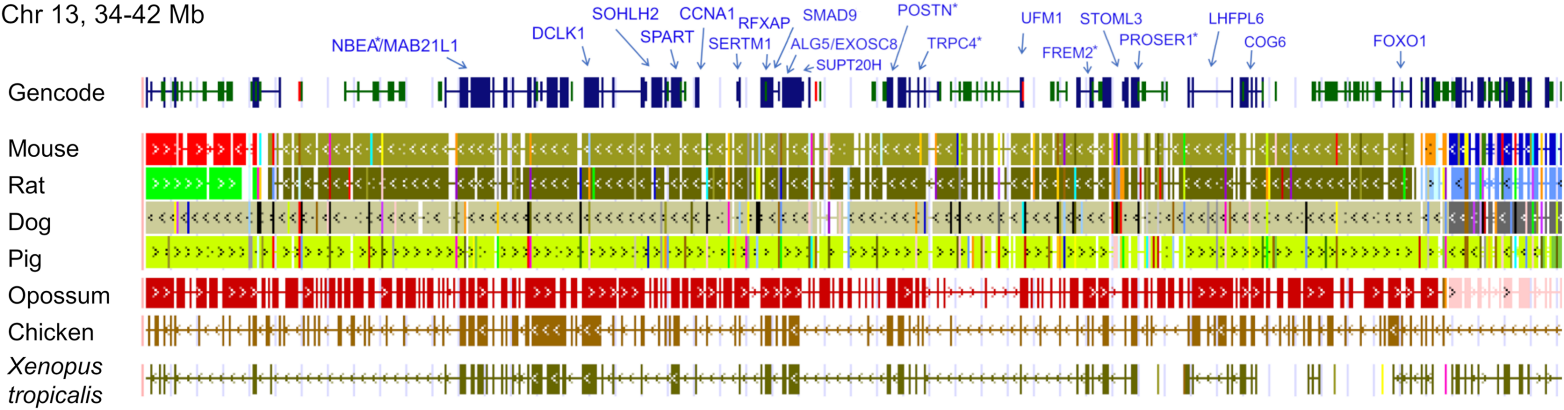
Demarcation of the NF synteny block based on gene order in human. Each row is a genomic data track derived from the University of California Santa Cruz (UCSC) Genome Browser, with the approximate location in the human genome indicated in the upper left. The top track shows ideograms of human genes curated by the Gencode consortium, indicating exon-intron structure and orientation, labeled with the gene symbols used in the text. Asterisks indicate genes with evidence of positive selection within the *Lasiurus cinereus* branch of the tested phylogeny. The subsequent tracks identify blocks of conserved sequence in representative Tetrapoda of increasing evolutionary distance to human. Within each species, alignments on the same chromosome share a common color and are linked by flow lines if contiguous. Regions that are approximately uniform in color and contiguous within each species are syntenic. Not the human gene *Ccdc169*, which lies between *Sohlh2* and *Spart*, is not conserved across mammals and therefore not labeled here. See Methods for data track sources.

The ten clustered selection candidates identified above combine two of three clusters identified in Cornman & Cryan (2022), which were on separate scaffolds of that assembly. They include four genes of the ‘cat-eye’ (CE) synteny block (*Cecr2*, *Cecr6*, *Mical3*, and *Slc25a18*) and three genes (*Postn*, *Frem2*, and *Proser1*) that lie within a second block of conserved synteny in tetrapods that was highlighted in Cornman & Cryan (2022). For convenience, I designate this latter synteny block “NF” based on the upstream and downstream genes *Nbea* and *Foxo1* that bound the protein-coding members of the block in human. The CE and NF synteny blocks are operationally defined here based on pre-computed alignments with other taxa (Figs. 2 and 3) available from the UCSC Genome Browser (Lee et al., 2020). One positive-selection candidate, *Sacs*, lies within the NF synteny block in most bat species examined but not in mammals generally (further discussed below). Another positive-selection candidate, *Amer3*, lies between these two synteny blocks in *L. cinereus* whereas the final selection candidate is downstream of the CE synteny block (*Fgf9*). Two positive-selection candidates from Cornman & Cryan (2022) that also mapped to this region, *Rps13* and *Necap1*, were excluded from this analysis as they are likely retrogenes: the coding sequences of the annotated genes occur on single exons and both have unannotated, multiexon alignments elsewhere in the genome. Note that these synteny-block depictions are necessarily human-centric based on the available data tracks and do not imply that no other genes are present in these regions in other lineages or that humans have retained all genes that were present in these synteny blocks in the common ancestor.

Tests of positive selection in the *L. cinereus* lineage with the expanded set of 13 bat taxa were again significant for all ten of the previously identified selection candidates (*P* < 0.01 after false-discovery correction; Table 1). Given the larger number of taxa available for analysis and the clear relevance of this region to adaptation in *L. cinereus*, I also tested the remaining genes of the CE and NF synteny blocks (Figs. 2 and 3). For these additional tests, *Nbea* and *Trpc4* were also significant for the *L. cinereus* branch after FDR correction at *P* < 0.01 (Table 2). Thus, a total of 12 genes within a 12-Mb genomic window were identified as candidates for positive selection within the *L. cinereus* branch in this updated assessment.

**Table 1:** Branch-specific tests of evolutionary rate for previously identified positive-selection candidates clustered in the hoary bat genome. Significant *P*-values are bolded. See text for details.

Gene	Synteny block	lnL: Model = 0, NSsites = 0	lnL: Model = 2, NSsites = 0	Adjusted *P*-value	Background ω	Foreground ω
*Amer3*		−11,606.9213	−11,599.1666	**0.0003**	0.3127	0.7241
*Cecr2*	CE	−17,895.0499	−17,887.2513	**0.0003**	0.2194	0.4779
*Cecr6*	CE	−6,567.5161	−6,557.1220	**0.0000**	0.1386	0.4092
*Fgf9*		−1,467.9643	−1,462.3278	**0.0021**	0.0030	0.3742
*Frem2*	NF	−41,738.7078	−41,719.1637	**0.0000**	0.1449	0.3532
*Mical3*	CE	−22,368.7987	−22,362.1366	**0.0008**	0.1672	0.3137
*Periostin*	NF	−8,688.5241	−8,679.9892	**0.0002**	0.0584	0.2468
*Proser1*	NF	−11,209.2924	−11,201.2627	**0.0002**	0.2019	0.6130
*Sacs*		−47,422.3021	−47,411.1321	**0.0000**	0.0832	0.1817
*Slc25a18*	CE	−2,305.1623	−2,296.6956	**0.0002**	0.1605	2.2338

**Table 2:** Branch-specific tests of evolutionary rate for other genes of the NF and CE blocks, as defined in the text, in the hoary bat genome. Significant *P*-values are bolded. See text for details.

Gene	Synteny block	lnL: Model = 0, NSsites = 0	lnL: Model = 2, NSsites = 0	Adjusted *P*-value	Background ω	Foreground ω
*Alg5*	NF	−3,167.7468	−3,167.7380	0.9541	0.1248	0.1395
*Atp6v1e1*	CE	−1,851.9959	−1,850.9754	0.2882	0.0496	0.0001
*Bcl2l13*	CE	−4,528.8733	−4,528.8732	0.9922	0.2803	0.2792
*Ccna1*	NF	−5,422.2964	−5,421.3659	0.3067	0.1379	0.2666
*Cog6*	NF	−6,466.0667	−6,465.4972	0.3977	0.0531	0.0973
*Dclk1*	NF	−6,276.2568	−6,274.3244	0.1052	0.0180	0.0547
*Exosc8*	NF	−2,588.5123	−2,585.7166	0.0413	0.0762	0.3830
*Foxo1*	NF	−6,126.2541	−6,125.6486	0.3944	0.0572	0.0994
*Hdhd5*	CE	−4,230.8031	−4,230.7411	0.7997	0.1590	0.1912
*Il17ra*	CE	−10,733.4234	−10,729.4470	0.0118	0.2544	0.4890
*Mab21l1*	NF	−1,957.4403	−1,957.3366	0.7415	0.0476	0.0302
*Lhfpl6*	NF	−2,999.7726	−2,999.5977	0.7094	0.0021	0.0001
*Nbea*	NF	−28,137.9727	−28,125.8943	**0.0000**	0.0332	0.1234
*Pex26*	CE	−4,080.0169	−4,079.8760	0.7330	0.1967	0.1444
*Nhlrc3*	NF	−3,773.5075	−3,773.3928	0.7415	0.3949	0.3239
*Rfxap*	NF	−2,809.1775	−2,808.5338	0.3909	0.1045	0.1798
*Smad9*	NF	−4,432.7381	−4,432.0203	0.3694	0.0257	0.0353
*Spart*	NF	−8,726.5589	−8,725.2287	0.2058	0.1288	0.2327
*Supt20h*	NF	−7,073.7274	−7,073.2486	0.4371	0.1244	0.1845
*Trpc4*	NF	−9,860.3126	−9,851.2214	**0.0001**	0.0656	0.2388
*Tuba8*	CE	−3,897.1940	−3,897.1896	0.9553	0.0144	0.0160
*Usp18*	CE	−3,896.5612	−3,895.6903	0.3148	0.2748	0.5061

**Figure 4:**
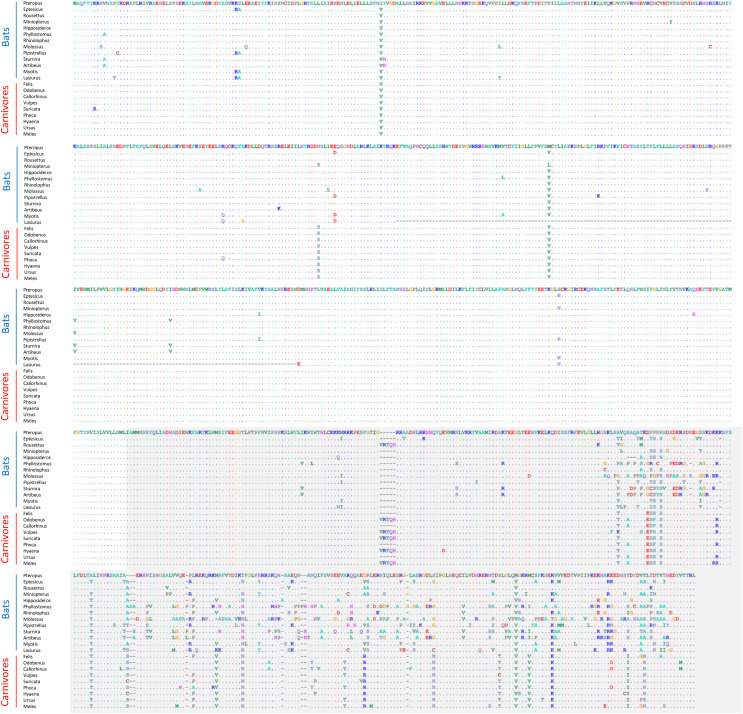
Alignment of predicted protein sequences of the Trpc4 gene in representative Carnivora and Chiroptera. The gray-shaded C-terminal region corresponds to the inositol triphosphate receptor (ITPR) binding region annotated in the human protein and discussed in the text. Residues that are unchanged from the first sequence in the alignment are represented by a dot to better highlight variable positions. Dashes indicate missing sequence. The alignment wraps to each row of the figure, position numbers are not shown for clarity.

None of these 12 alignments were significant for positive selection within bats generally (*i.e*., testing a site-level model with no branch-specific parameters) after FDR correction (File S2). However, the *Trpc4* alignment was observed to be highly variable in the C-terminal third of the coding sequence within bats overall as well as divergent from the other tetrapod clades in the same region (Fig. 4). In contrast, other tetrapod orders did not show increased variation in this region either within or among clades. *Trpc4* encodes a Ca^2+^ transmembrane channel subunit involved in diverse processes, the C-terminal region of which is intracellular and believed to interact with inositol triphosphate receptors (ITPRs) as well as calmodulin (Tang et al., 2001). Given the diverse hibernation strategies of bats, it is noteworthy that *Trpc4* is required in mice for heat detection and subsequent thermoregulation by warm-sensing neurons (Zhou et al. 2023). I therefore performed additional *post-hoc* tests of positive diversifying selection on *Trpc4* within bats relative to a representative mammalian outgroup, Carnivora. With *Trpc4* sequences from carnivores designated as background and bat sequences designated as foreground, PAML analysis revealed strong evidence of diversifying selection within bats for the entire coding sequence (1.12E-7) and for the ITPR-binding domain alone (3.36E-7). Remarkably, neither BUSTED nor aBSREL supported positive selection in those same alignments. This discrepancy might be attributable to the greater evolutionary distance between bats and carnivores, which could complicate the estimation of background evolutionary rates due to factors such as rate variability and mutational saturation (reviewed in Joy et al., 2016). Alternatively, the ITPR region may simply be evolving neutrally within bats, yet this seems unlikely given that truncations and frameshifts are not seen and radical substitutions are infrequent (Fig. 4). Factors such as gene conversion or unrecognized paralogy also do not appear to contribute to the observed *Trpc4* diversity, as BLASTP searches with either the full *P. kuhli* predicted protein or the ITPR region alone did not match more strongly to any other Trpc homolog (File S2). Alterative splicing can also be excluded as a confounding factor as the predicted exon structures are similar to orthologs in other taxa and an outgroup sequence from cat aligned only to predicted exons in *P. kuhlii* (see Fig. S3).

### Structural evolution of the CE and NF synteny blocks in bats and tetrapod outgroups

**Figure 5:**
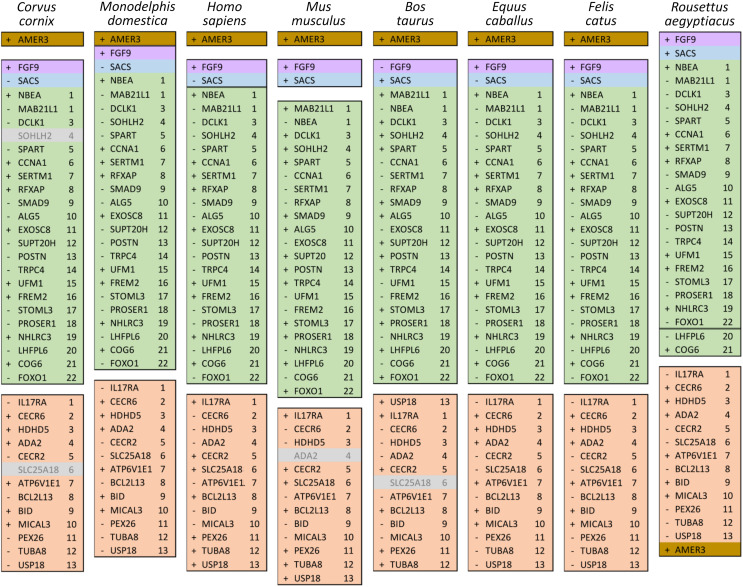
Relative positions in tetrapod genomes of synteny blocks and individual genes analyzed in this study. Each species diagram consists of one gene per row, represented by the human gene symbol for the orthologous group. A plus or minus sign indicates the orientation of the gene on the reference sequence. Gaps between gene blocks indicate they are on different linkage groups. A double line between genes on the same linkage group indicates a gap greater than 10 Mb. The *Nbea*-*Foxo1* (NF) synteny block is colored green and the cat-eye (CE) synteny block is colored orange (see text for definitions of these blocks). Within each block, genes are numbered according to their order in the human genome as a reference. The genes *Sacs*, *Fgf9*, and *Amer3* are not numbered because they are not considered part of either synteny block; rather, they are shown because they were identified as positive selection candidates closely linked to the two synteny blocks in *Lasiurus cinereus*. These three genes are colored blue, purple, and brown, respectively. Gene symbols are in unitalicized upper case for legibility. Genes that are unannotated and presumed absent in a species are grayed.

**Figure 6:**
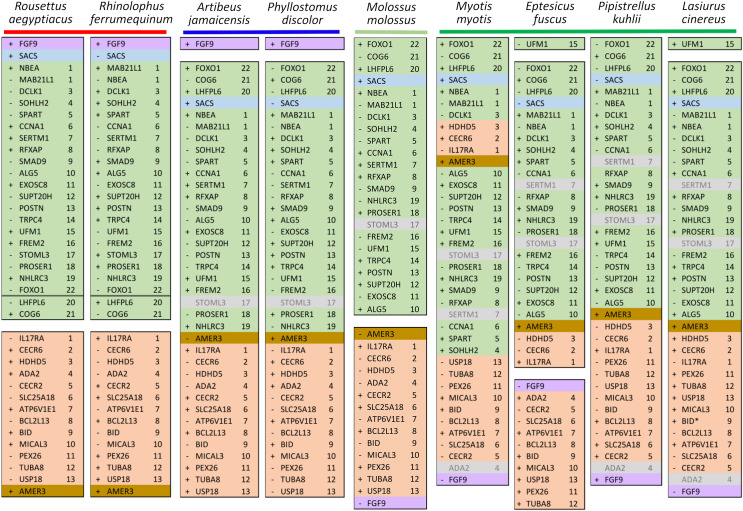
Relative positions in twelve tetrapod genomes of synteny blocks and individual genes analyzed in this study. Each species diagram consists of one gene per row, represented by the human gene symbol for the orthologous group. A plus or minus sign indicates the orientation of the gene on the reference sequence. Gaps between genes indicate they are on different linkage groups. A double line between genes on the same linkage group indicates a physical distance greater than 10 Mb. The *Nbea*-*Foxo1* (NF) synteny block is colored green and the cat-eye (CE) synteny block is colored orange (see text for definitions of these blocks). Within each block, genes are numbered according to their order in the human genome as a reference. The genes *Sacs*, *Fgf9* and *Amer3* are not numbered because they are not considered part of either synteny block; rather, they are shown because they were identified as positive selection candidates closely linked to the two synteny blocks in *Lasiurus cinereus*. These three genes are colored blue, purple, and brown, respectively. Gene symbols are in unitalicized upper case for legibility. Genes that are unannotated and presumed absent in a species are grayed. Species are grouped by phylogenetic position using the same color scheme as in Fig. 7. The asterisk denotes uncertainty as to whether the gene *Bid* is functional in *L. cinereus*.

Ten tetrapod taxa including a bird, non-placental mammals, and placental mammals were surveyed to evaluate the ancestral organization of the clustered positive selection candidates and the tempo of structural change in their vicinity during tetrapod evolution (File S3), a representative subset of which is shown in Fig. 5. Some structural variation in the CE block is seen in the *B. taurus* and *O. anatinus* genomes and individual gene deletions have occurred (*e.g*., *Ada2* in *M. musculus* and *Slc25a18* in *S. scofra* and *B. taurus*). However, altered gene order or orientation was not observed in the NF block nor is it linked with the CE block in any outgroup genome. The bats *R. aegypticus* and *R. ferrumiquinum*, representatives of suborder Yinpterochiroptera (Agnarsson et al., 2011; Amador et al., 2018), have genomic architectures (Figs. 5 and 6) similar to the other tetrapod clades, but differ in that *Lhflp6* and *Cog6* are far removed from other NF genes on the same linkage group. The positively selected gene *Amer3* does not occur near either the NF or CE synteny blocks in other tetrapods but is linked to CE genes in all bats examined. The positively selected genes *Sacs* and *Fgf9* have a conserved relative orientation in outgroups and while usually linked to the NF synteny block they never lie within either synteny block (unlike in bats).

**Figure 7:**
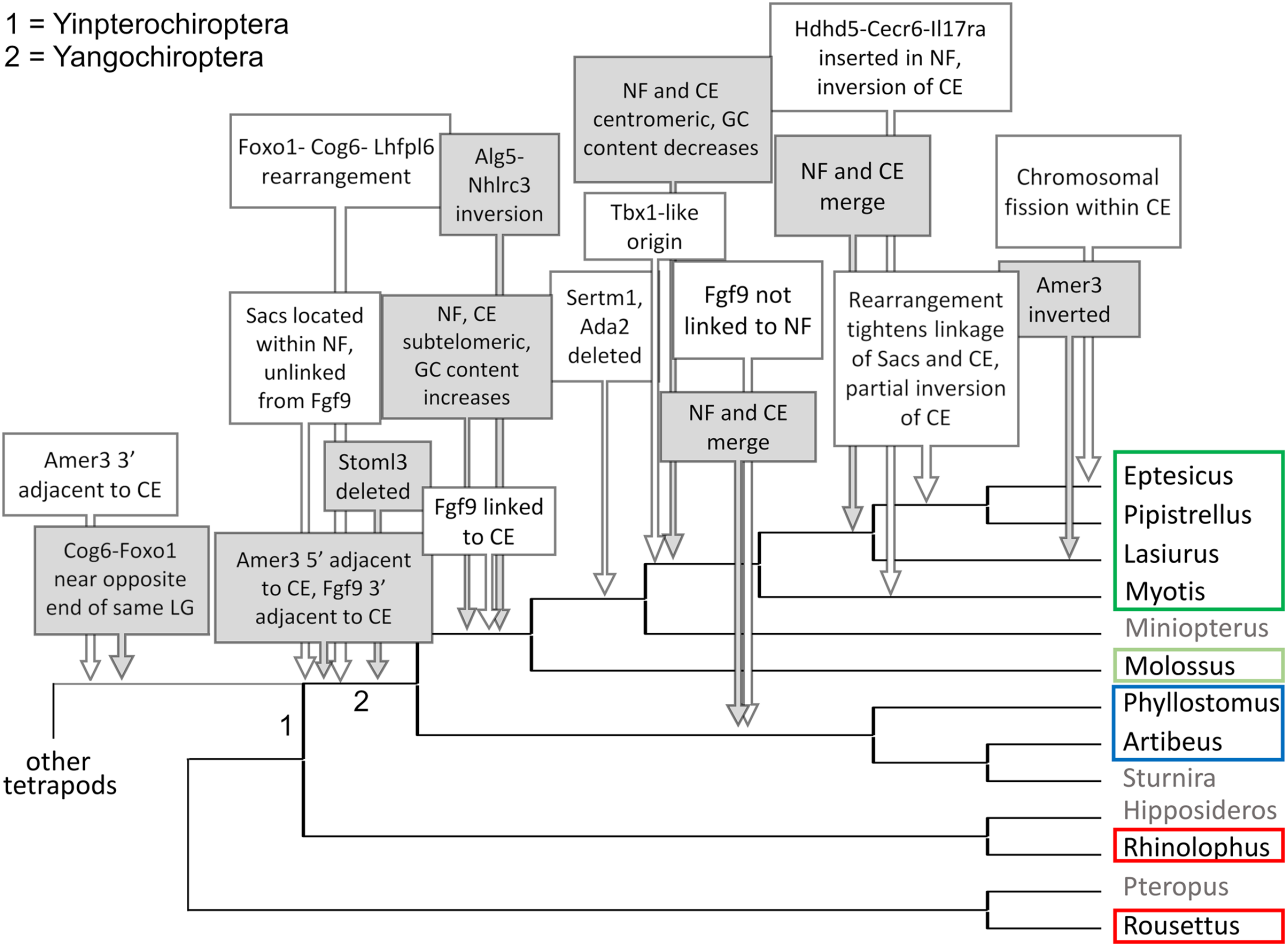
Hypothesized sequence of structural changes in gene organization in bats. Taxa for which chromosome-scale assemblies were available for this analysis are marked by colored boxes, which correspond to the colors used in Fig. 6. Grayed taxa were not analyzed for synteny because the relevant genes were not on large linkage groups. Genes are identified by their gene symbols, whereas NF and CE denote synteny blocks described in the text. GC denotes G + C content of gene coding sequences.

Within Yangochiroptera (Fig. 6 and File S3), multiple distinct arrangements are evident within and among the CE and NF synteny blocks, as well as the positively selected genes *Sacs*, *Fgf9*, and *Amer3*. A common arrangement is shared between *P. discolor* and *A. jamaicensis*, of family Phyllostomidae (Yangochiroptera), in which the NF and CE blocks are tightly linked, the NF block is further rearranged in gene order, *Amer3* lies between *Il17ra* and *Nhlrc3*, and *Fgf9* is effectively unlinked from *Sacs* and genes of both synteny blocks. Organization of these gene regions is more variable within the four vesper bats examined, such that a single unambiguous sequence of chromosomal rearrangements is not apparent without homoplasy (Fig. 7 illustrates one possible reconstruction of evolutionary events, inferred by inspection). The vespertilionid species share at least partial linkage of NF and CE genes (Fig. 6, File S3), but in arrangements distinct from phyllostomids. In *E. fuscus*, only the CE genes *Il17ra*, *Cecr6*, and *Hdhd5* are linked with NF, with the remainder on a separate linkage group, apparently due to a chromosomal fission. *Amer3* again lies between the two synteny blocks, but in two novel arrangements in vesper bats. *M. myotis* exhibits a unique integration of the *Il17ra*-*Cecr2*-*Hdhd5* trio and *Amer3* within the NF block. *P. kuhlii* and *L. cinereus* are more similar in gene order compared with other vesper bats examined, but differ in that *Ufm1* of the NF synteny block is on a different scaffold in *L. cinereus* (it also has a distinct location in *E. fuscus*) and *Amer3* is in the same relative order but inverted in *L. cinereus*. Note that the ideograms in Figs. 5 and 6 are oriented with the NF block first and the CE block second for consistency, since the plus-strand designation of each linkage group is arbitrary (hence *P. kuhlii* and *L. cinereus* coordinates are descending and ascending, respectively, on the main linkage groups; see File S3 for genomic accessions and coordinates).

The genomic organization in *M. molossus*, representative of the free-tailed bat family (Molossidae), reveals several changes from Yinpterochiroptera that are shared with phyllostomid and vespertilionid bats and may be basal to Yangochiroptera: 1) the insertion of *Sacs* within the NF synteny block; 2) deletion of *Stoml3*; and 3) a rearrangement of the NF genes *Lhfpl6*-*Foxo1*. However, the consensus view that Molossidae is within the superfamily Vespertilionoidea (Agnarsson et al., 2011; Amador et al., 2018) and shares a more recent common ancestor with vespertilionids than with phyllostomids is incongruent with aspects of Fig. 6. Most notably, this phylogenetic position implies that either the NF and CE blocks merged independently in vespertilionid and phyllostomid ancestors, or the merged NF and CE blocks subsequently split in molossids (a reversal). Further complicating the inferred order of chromosomal changes, the molossid and phyllostomid taxa also share a very pronounced increase in GC content within the coding sequences of the analyzed genes (Fig. S4). This shift in background nucleotide composition may relate to the extreme subtelomeric location of NF and CE genes in some of these taxa. For example, the most 3’ gene coordinate of the merged NF+CE block in *A. jamaicensis* is only six bases from the end of the linkage group (File S3). Changes in genomic location relative to chromosome ends are known to influence background nucleotide composition in mammals, and these compositional shifts can occur rapidly (Montoya-Burgos, Boursot & Galtier, 2003).

While Figs. 5 and 6 illustrate the changing relative positions of the positive selection candidates *Amer3*, *Fgf9*, and *Sacs* in tetrapod genomes, it should not be inferred that those genes have necessarily moved individually. Identifying and plotting conserved landmark genes that are adjacent to those positive selection candidates in outgroup and bat genomes demonstrates that each of the three selection candidates has moved as part of larger multigene blocks (Figs. S5 and S6). *Amer3* likely moved as part of an ancestral block of eight genes (Fig. S5), whereas the genes that flank them in other mammals, represented by the human gene order in Fig. S5B, were not lost in bats but are instead located distant from *Amer3* on the same chromosome or on a different chromosome. Additional inversions of these discrete gene blocks have subsequently occurred during bat evolution, giving rise to diverse relative orientations (Fig. S5). Linkage of the *Amer3* block to the CE and NF genes has also been maintained despite subsequent chromosomal breakage (*e.g*., the genes are telomeric in *A. jamaicensis* and *M. molossus* as noted above but occur in the middle of large linkage groups in the other taxa shown in Fig. S5).

The selection candidates *Fgf9* and *Sacs* are tightly linked in all outgroup genomes examined (Fig. S6) but based on conserved landmark genes have split into two distinct, rearranged blocks in bats. (Note other, less conserved genes are also present in the vicinity but are not shown for clarity.) These gene blocks are maintained in approximately the same order in outgroups, with the exception of an inversion in mouse, and lie several Mb from the NF block. In bats, the *Fgf9* and *Sacs* blocks are separated from each other by several Mb or are on separate linkage groups. In molossid and vespertilionid bats, the Fgf9 block is tightly linked to the NF block and has remained so through subsequent rearrangements in the region. The *Fgf9* block has also remained largely intact in all bat lineages examined, whereas most genes of the *Sacs* block present in Yinpterochiroptera are found elsewhere in the genomes of Yangochiroptera.

It remains to be determined whether the CE gene *Bid* is intact in *L. cinereus*. BLASTN alignments of *Bid* orthologs from other Vespertilionidae identified only a single contiguous match of ~200 nt within the *L. cinereus* assembly (for comparison, the coding sequence is ~700 nt in *E. fuscus*). Gene pseudogenization and loss are themselves functionally significant events that may relate to adaptive divergence as well, and indeed three genes of the NF and CE blocks (*Ada2*, *Stoml3*, and *Sertm1*) were not found in multiple vespertilionid bats. Pseudogenization of at least three genes has also substantially altered the *Amer3* block subsequent to being linked to the NF and CE blocks in bats, although *Gpr148* has been lost in other mammals as well (Fig. S5A). However, some presumed gene losses could simply reflect assembly errors, such that transcriptomic data may be needed to confirm their sequence and functionality. A summary of gene order and orientation of all gene blocks discussed here, including gene loss events, is depicted for bats and outgroups in Fig. S7.

### Functional roles of positively selected genes and synteny blocks

Eight of the 12 positive selection candidates in *L. cinereus* have peak expression in brain tissue in either human or mouse based on NCBI Gene data, whereas nine have enriched expression in a brain tissue category according to the DAVID “Up_tissue” annotation table (Table 3). Similarly biased expression is seen in the DAVID data when all genes in the NF and CE blocks are included regardless of selection test result. For the 38 genes for which DAVID annotation information was available, 29 (76.3%) had elevated expression in human brain tissue (Table 3). However, a smaller proportion of genes (13 of 37 genes with available data, or 35.1%) had peak expression in brain or central nervous system (CNS) in either human or mouse based on NCBI Gene data. No significant gene ontology enrichment was found for either the selected genes or for all NF and CE genes. I conclude that while the positive selection candidates have relatively high expression in brain or CNS, as do the NF and CE genes as a group, neither gene set is over-represented in annotated pathways or biological processes. Furthermore, a recent protein-protein interaction map of the mouse brain uncovered no direct pairwise interactions between proteins encoded by the genes listed in Fig. 5 (see Table S3 of Pourhaghighi et al., 2020).

**Table 3:** Tissue-specific expression patterns for positive-selection candidates as well as other genes of the NF and CE blocks (see text for block definition). The sources of tissue-specific expression are described in the text. Tissues that are part of the brain or central nervous system are bolded. Tissue labels that are specific to human disease states were excluded. Positive selection candidates are denoted by an asterisk.

Gene symbol	Gene name	DAVID tissue enrichment	NCBI tissue of peak expression (human/mouse)
*Ada2*	Adenosine deaminase 2	**Brain**, Liver, Thymus, Trachea, Uterus	Spleen/NA
*Alg5*	Dolichyl-phosphate beta-glucosyltransferase	**Hypothalamus**, Liver, Prostate, Umbilical cord blood	Thyroid/placenta adult
Amer3*	APC membrane recruitment protein 3	**Brain, Cerebellum**	**Brain/CNS E18**
*Atp6v1e1*	ATPase H+ transporting V1 subunit E1	**Amygdala**, **Brain**, **Cajal-Retzius cell**, **Cerebellum**, Liver, Lung	**Brain/cortex adult**
*Bcl2l13*	BCL2 like 13	**Amygdala**, **Brain**, Eye, Human skeletal muscle, Liver, Skin, Synovial membrane tissue, Testis, Trachea	Fat/heart adult
*Bid*	BH3 interacting domain death agonist	**Brain**, Embryonic kidney, Fetal liver, Liver, Normal colorectal tissue, Skin	Bone marrow/kidney adult
*Ccna1*	Cyclin A1	**Brain**, Myeloid, Testis	Testis/testis adult
Cecr2*	Histone acetyl-lysine reader	**Brain**, Liver, Skeletal muscle	**Brain**/testis adult
Cecr6*	Transmembrane protein 121B	**Brain**	**Brain**/NA
*Cog6*	Component of oligomeric golgi complex 6	**Amygdala**, Aorta, **Brain**, Fetal skin	Testis/testis adult
*Dclk1*	Doublecortin like kinase 1	**Brain, Fetal brain, Hippocampus**	**Brain/frontal lobe adult**
*Exosc8*	Exosome component 8	**Brain**, Uterus	Testis/**CNS E11.5**
Fgf9*	Fibroblast growth factor 9	Foreskin, Kidney	Kidney/**cerebellum adult**
*Foxo1*	Forkhead box O1	Lymph, Placenta	Ovary/ovary adult
Frem2*	FRAS1 related extracellular matrix 2	Fetal kidney, Plasma, Tongue	Kidney/bladder adult
*Hdhd5*	Haloacid dehalogenase like hydrolase domain containing 5	**Brain**, Embryo, Lymph, Testis	Duodenum/thymus adult
*Il17ra*	Interleukin 17 receptor A	Placenta, T-cell, Uterus	Bone marrow/thymus adult
*Lhfpl6*	LHFPL tetraspan subfamily member 6	**Amygdala**, Lung	Fat/lung adult
*Mab21l1*	Mab-21 like 1	**Brain**	NA/NA
Mical3*	Microtubule associated monooxygenase, calponin and LIM domain containing 3	**Brain**, Liver, Lymph, Pancreas, Testis	Testis/testis adult
Nbea*	Neurobeachin	**Brain**, Embryonic head, Spleen, Testis	**Brain/CNS E18**
*Nhlrc3*	NHL repeat containing 3	Heart, Placenta, Testis	Thyroid/placenta adult
*Pex26*	Peroxisomal biogenesis factor 26	**Brain**, Colon, Fibroblast, Ileal mucosa, Uterus	Colon/ovary adult
Postn*	Periostin	Liver, Periodontal ligament, Placenta, Plasma, Thyroid gland	Skin/limb E14.5
Proser1*	Proline and serine rich 1	**Amygdala, Brain**, Colon endothelium, Peripheral nervous system	Placenta/thymus adult
*Rfxap*	Regulatory factor X associated protein	Lymphoblast, Testis, **Thalamus**	Testis/ovary adult
Sacs*	Sacsin	**Astrocyte, Brain**, Fetal liver, Uterine endothelium	**Brain/CNS E18**
*Sertm1*	Serine rich and transmembrane domain containing 1	**Amygdala, Brain**	**Brain/CNS E18**
Slc25a18*	Solute carrier family 25 member 18	**Brain**, Liver	**Brain/cortex adult**
*Smad9*	SMAD family member 9	**Brain**, Eye, **Fetal brain**	Thyroid/adrenal adult
*Sohlh2*	Spermatogenesis and oogenesis specific basic helix-loop-helix 2	Testis	Testis/testis adult
*Spart*	Spartin	**Brain**, Placenta	Ovary/limb E14.5
*Stoml3*	Stomatin like 3	Lung, Trachea	Lung/**frontal lobe adult**
*Supt20h*	SPT20 homolog, SAGA complex component	Kidney, Prostate, Testis, Trachea	Testis/testis adult
Trpc4*	Transient receptor potential cation channel subfamily C member 4	Embryonic kidney, Kidney, **Thalamus**	Endometrium/**frontal lobe adult**
*Tuba8*	Tubulin alpha 8	**Amygdala, Brain**, **Caudate nucleus**, Skeletal muscle	Heart/testis adult
*Ufm1*	Ubiquitin fold modifier 1	Bone marrow, **Brain**, Kidney	Thyroid/placenta adult
*Usp18*	Ubiquitin specific peptidase 18	**Brain**, Ovary, Uterus	Fat/liver E18

For five selection candidates, deleterious mutations are associated with mild to severe defects of organogenesis or embryonic neural development, as indicated by clinical variants in human or by experiments in animal models. *Cecr2* deletion causes anencephaly, a severe defect of cranial and neural development (Banting et al., 2005; Fairbridge et al., 2010; Dicipulo et al., 2021). *Frem2* loss of function underlies Fraser Syndrome, characteristics of which include cryptopthalmy and syndactyly, both of which can be recapitulated in a mouse model (Jadeja et al., 2005; Timmer et al., 2005). A frameshift in *Proser1* has been associated with craniofacial dysmorphy and genitourinary developmental defects in human (Salah et al., 2022). Missense mutations in *Fgf9* are associated with multiple syntoses syndrome, which is characterized by joint fusions of the hand and cranium as well as craniofacial dysmorphy (Wu et al., 2009; Rodriguez‐Zabala et al., 2017); these phenotypes can be recapitulated in a mouse model (Tang et al., 2017). Missense mutations, microdeletions, and reciprocal translocations in *Nbea* are associated with neurodevelopmental disease, including autism and epilepsy (Mulhern et al., 2018). While no developmental defect has been reported for *Amer3* specifically, the homolog to which *Amer3* binds, *Amer1*, does have an association with craniofacial dysmorphy and abnormal organogenesis in human (Mi et al., 2020). However, not all deleterious phenotypes of selection candidates appear early in development: *Sacs* mutations underlie an adult-onset neurodegenerative syndrome characterized by spastic ataxia (Bagaria, Bagyinszky & An, 2022).

In addition to these gene-specific associations, diverse structural aberrations in human involving the CE block, such as supernumerary chromosomes and microdeletions, have overlapping phenotypes with a craniofacial component (reviewed in Glaeser et al., 2021). A spontaneous deletion of the majority of NF genes has been associated with impaired neurological development and craniofacial dysmorphy in an isolated clinical report (Miura et al., 2020). Recurrent deletion or duplication of five genes that include *Amer3* (also known as *Fam123C*) is associated with clinically diagnosed behavioral problems, epilepsy, and cranial dysmorphy (Dharmadhikari et al., 2012); the deletion spans the genes *Gpr148* to *Plekthb2* shown in Fig. S5A.

The phenotypes of deleterious mutations reveal aspects of gene function, but may be completely unrelated to positively selected phenotypic variation. Nonetheless, bats are well known for extreme cranial divergence that underpins ecological traits. Cranial morphology has been shown to evolve *via* allometric processes such as heterochrony (Camacho et al., 2020), the underlying mechanisms of which are beginning to be revealed (Camacho et al., 2019). Arbour, Curtis & Santana (2021) identified distinct modules of cranial and mandibular development that have diversified across the bat phylogeny, particularly with respect to oral-echolocating, nasal-echolocating, and non-echolocating taxa. The biomechanics of feeding also strongly shapes cranial evolution at both deep and shallow divergence times (Hedrick & Dumont, 2018; Camacho et al., 2019). For example, cranial morphology was found to parallel dietary similarity in some Mediterranean *Myotis* species (Evin et al., 2008).

### Birth and death of a Tbx1-like gene family in Vespertilionidae

Cornman & Cryan (2022) identified a variable gene family with partial homology to the DNA-binding transcription factor *Tbx1*, the latter having many important roles in embryonic development (Baldini, Fulcoli & Illingworth, 2017). The gene family was initially identified because one member lies within the CE block in *L. cinereus* but homologs were not detected in other tetrapod orders. In addition to multiexon gene models, single-exon genes and partial genes were identified, suggesting that at least some of these ‘*Tbx1*-like’ family members were retrogenes or pseudogenes. To further investigate the origins and functionality of this gene-family expansion, I performed a TBLASTN search with the homologous sequence XP_027987819.1 from *E. fuscus* against the genomes of *P. kuhlii* and *M. myotis*. Multiple unannotated *Tbx1*-like homologs are present in both of the latter genomes (File S4), at least some of which appear to have the minimum complement of gene features (a multiexon example with a predicted core promoter is shown in File S5). No Tbx1-like sequences were identified in non-vespertilionid bat genomes.

High-scoring BLAST matches in *P. kuhlii* are also consistently supported by low-level RNA-Seq coverage at those sites (Fig. S8B). Furthermore, the well conserved portion of the *Tbx1*-like family retains the key arginine residue that in the TBX1 protein binds the major groove of double-stranded DNA (El Omari et al., 2012), and the N-terminal protein regions have secondary structures very similar to that of human *Tbx1* even after all primary sequence homology is lost (Fig. S8A). In contrast, the C-terminal portions of the predicted proteins appears more disordered and lack the second and third alpha helices and DNA interaction residues found in TBX1. This pattern of sequence divergence also characterizes the *Tbx* gene family generally (El Omari et al., 2012), in that the conserved protein domain that defines the *Tbx* family encompasses the same region that *Tbx1* shares with *Tbx1*-like, whereas *Tbx* homologs show increased divergence at the same point that *Tbx1*-like diverges from *Tbx1*, which encompasses the dimerization domain.

Evidence of transcription and conservation of functionally important domains suggests that at least some *Tbx1*-like genes are functional, albeit with potentially high evolutionary turnover or pseudogenization rates. The latter possibility is illustrated by a comparison of the *P. kuhlii Tbx1*-like gene proposed in File S5 to its closest homologs in three other vespertilionids, including *L. cinereus* (Fig. S8C). All three homologs in these other taxa show clear signs of pseudogenization, including internal stop codons, frameshifts, and an unconserved start codon. I conclude that the burst of *Tbx1*-like sequences began early in the Vespertilionid lineage if not earlier, likely with continued birth and death in subsequent lineages.

While pseudogenes are ubiquitous in complex genomes (and often disregarded on non-scientific grounds (Cheetham, Faulkner & Dinger, 2020), the apparent burst in *Tbx1*-like duplications within the same lineage exhibiting positive selection on genes affecting cranial morphology would be a remarkable coincidence if functionally unrelated. This is because *Tbx1* mutation, duplication, and haploinsufficiency are all associated with craniofacial dysmorphy syndrome in human (DiGeorge Syndrome), which can be recapitulated in a mouse model (Lindsay et al., 2001; Liao et al., 2004; McDermid & Morrow, 2002). An expansive literature has since established *Tbx1* as a key regulator of cranial development, governing aspects of the differentiation and migration of cells arising from the pharyngeal arches and neural crest (reviewed in Baldini, Fulcoli & Illingworth, 2017). The human phenotypes associated with *Tbx1* dosage variation overlap with cat-eye syndrome and both syndromes are attributable in part to defects in pharyngeal arch development mediated by abnormal cell migration (Tan et al., 2010, Ivins & Scambler, 2022). Indeed, *Tbx1* is tightly linked to the CE synteny block in human and deletions of either constitute similar subclasses of 22q11 microdeletion syndrome (Tan et al., 2010), although *Tbx1* is not located near either synteny block in mammals generally (see *Tbx1* gene pages for taxa in Fig. 5 and File S3). One hypothesis suggested by the advent of *Tbx1*-like sequences in vesper bats is that they have been at least transiently functional and have affected aspects of *Tbx1*-regulatory networks relevant to positively selected phenotypes in the hoary bat lineage, perhaps by modulating or inhibiting TBX1 dimerization at particular binding sites. Tbx1 is believed to regulate gene expression by binding C-rich DNA motifs and then promoting histone methylation near transcription start sites in a dosage-dependent fashion (Fulcoli et al., 2016). This hypothesis does not require that TBX1 directly regulate selection candidates or other genes with which they are linked, although 3 of 12 selection candidates (*Frem2*, *Proser1*, and *Slc25a18*) were differential expressed in a mouse *Tbx1* haploinsufficiency model (Fulcoli et al., 2016), compared with 1,992 of 22,807 total genes (8.7%) tested in that study.

## Caveats and conclusions

This analysis further established a cluster of 12 genes exhibiting signatures of diversifying selection within the hoary bat lineage, which lie within a 12-Mb window of the hoary bat genome. This genomic hotspot is dominated by re-assorted elements of two distinct synteny blocks that are conserved and unlinked in other tetrapods. In fact, structural changes within this region during bat evolution appears to require parallel occurrences or subsequent reversals in different bat lineages. The selected genes specifically and the synteny blocks generally are associated with cranial and neural development, based on expression patterns, disease associations, and functional studies in model organisms. The analysis also confirmed previously reported *Tbx1*-like duplications within vesper bats, although the timing remains difficult to determine since the sequences are largely unannotated in those genomes and turnover appears rapid. Nonetheless, the strict conservation of a critical functional region despite high divergence elsewhere in the predicted proteins (see also Cornman & Cryan, 2022) implies purifying selection. I conclude that this genomic region is a hotspot of adaptive evolution in the hoary bat lineage that likely relates to cranial and neurological traits underlying ecological diversification.

Different tests of selection fit conceptually distinct, parameter-rich models to quantify excess nonsynonymous substitutions in an alignment, a phenomenon that is likely both transient and conservative as a metric of positive selection (see Seo, Kishino & Thorne, 2004). Not surprisingly, different algorithms and data sets give different results. For example, positive rates were higher with the PAML package than with the HyPhy package, and the PAML algorithm is known to be subject to false positives if the assumption of rate homogeneity on background lineages is unreasonable (Smith et al., 2015). Nonetheless, results from the two packages were broadly similar for longer alignments, indicating convergence of model results as the information content of alignments increased. For example, using either tree 1 or tree 2, six of nine alignments exceeding 2,000 analyzed positions were supported by the BUSTED method at an FDR-corrected *P*-value of less than 0.05 (File S2). For tree 3, seven of nine were concordant. Incomplete concordance between the two methods may also arise if diversification had begun prior to the divergence of *Lasiurus*, as suggested by the fact that the aBSREL method was generally not significant for the same comparisons. Methodological congruence might therefore be greater for alternative designations of foreground branches. Yet the diversification of *Trpc4* within bats (Fig. 4) is a striking example of how different approaches to quantifying episodic selection can produce highly discordant interpretations of the same alignment.

Another caveat is that evolutionary rate estimation may be sensitive to errors in phylogeny or reconstructed states at unsampled nodes (Feng et al., 2020) and factors such as undetected paralogy, gene conversion, nucleotide composition bias, and recombination can lead to false positives (Anisimova, Nielsen & Yang, 2003; Galtier & Duret, 2007; Ratnakumar et al., 2010). For example, two genes identified as positive selection candidates in this region by Cornman & Cryan (2022), *Rps13* and *Necap1*, were found to be paralogous retrogenes and removed from this analysis for this reason. Phylogenetic uncertainty also exists for the studied species, particularly with respect to vespertilionid taxa. This uncertainty was addressed by evaluating alternative guide trees, which produced qualitatively similar PAML results. Pruning compositionally skewed taxa (tree 4) generally increased *P*-values of the tests such that fewer were significant at the same alpha, yet most remained significant at an FDR-corrected *P*-value of 0.05. Furthermore, the closest outgroups of *L. cinereus* are very similar in nucleotide composition of tested genes (Fig. S3) and thus unlikely to bias the *L. cinereus* branch test. Moreover, the shift in background composition affected whole genomic regions yet only a minority of tested genes within them had signatures of positive selection.

Despite these important caveats, it bears emphasizing that the conclusions of this study are not predicated on any specific gene undergoing episodic positive selection. They are instead based on the tight genomic clustering of numerous positive selection tests concomitant with a high number of structural changes within synteny blocks that show few changes in other tetrapod orders. These observations hold regardless of any methodological sensitivity for a specific gene. This study also strengthens the genome-wide conclusions of Cornman & Cryan (2022), given that all ten positive selection tests repeated here with additional data produced the same result at an alpha of 0.01. That study also identified several positively selected genes affecting cranial development elsewhere in the *L. cinereus* genome.

This study examined the order and evolutionary rates of protein-coding genes only. Purifying selection on the expression context of long noncoding RNA (lncRNA) genes has been proposed to constrain coding-gene synteny in some cases (Hufton et al., 2009), and lncRNA genes occur within these synteny blocks in many taxa. For example, the human genes *Cecr1*, *Cecr7*, and *Fam230D* are all lncRNAs within the CE block defined by Fig. 1. However, *Cecr1* and *Cecr7* have been shown not to be conserved with other mammals (*e.g*., Bridgland et al., 2003; Footz et al., 2001) and both *Cecr7* and *Fam230D* appear to be recently arisen within the primate lineage (Bridgland et al., 2003; Delihas, 2018). lncRNA genes present in the human NF synteny block (*e.g*., *LINC02343* and *LINC00571*) also do not have conserved orthologs listed in the UCSC genome browser. While the annotation of lncRNAs remains poorly developed compared with protein-coding genes, conservation of orthologous lncRNAs *per se* does not appear to drive the maintenance of the NF and CE blocks across tetrapod orders. Yet regulation of chromosomal ‘neighborhoods’ (*sensu* Nora, Dekker & Heard, 2013) by lncRNAs could still be a mechanism by which synteny is maintained even if the lncRNAs themselves turn over or no longer retain evidence of orthology (Engreitz et al., 2016; Quinn et al., 2016).

Regardless of the contribution of lncRNAs, co-regulation *via* shared *cis* regulatory elements and chromatin neighborhood effects remains an important hypothesis for the maintenance of these synteny blocks during tetrapod evolution as well as the clustering of positive selection candidates in *L. cinereus*. For example, ‘transcriptionally associated chromosome domains’ are recognized facets of hierarchical chromatin folding that contribute to correlated transcription on scales from kilobases to megabases (Nora, Dekker & Heard, 2013), distances that are very relevant to the synteny blocks studied here. Moreover, the critical link between chromatin state and co-expression may be manifested in only a few cell types or developmental stages (cf. Eckalbar et al., 2016), and thus not apparent in aggregate measures of gene expression such as given in Table 3.

Future directions could include refining estimates of the timing and magnitude of episodic selection within clades as more genomes become available. The hypothesis that structural changes within synteny blocks alter gene expression profiles, chromatin modifications, or chromatin topological domains could potentially be tested with RNA-Seq, ChIP-Seq, and HiC data, although the fact that many of these genes act during embryonic development is constraining (but see Eckalbar et al., 2016 for an example in bats). Fine-scale analysis of genotype-phenotype associations for these genes, *e.g*., by focusing on highly diverse genera such as *Myotis*, could suggest ecological drivers of diversifying selection. Further comparative genomic study of the orthologous genes in bats could also serve as a case study of how genic and karyotypic evolution interact to drive phenotypic divergence, given that karyotypic evolution is considered an important mode of adaptation and species diversification (Damas, Corbo & Lewin, 2021).

## Supplemental Information

10.7717/peerj.17482/supp-1 Supplemental Information 1 Phylogenetic topologies used as guide trees for evolutionary analysis. Tree 1 is consistent with Amador et al. (2018). Tree 2 differs from tree 1 only in postulating that Lasiurus is closer to Pipistrellus than the latter is to Eptesicus. Tree 3 is consistent with Agnarsson et al. (2011). Tree 4 is the same as Tree 1 except that four taxa with skewed nucleotide composition have been pruned (see text for details). <v:shape id="Picture_x0020_15" o:spid="_x0000_i1025" type="#_x0000_t75" alt="Diagram Description automatically generated"> <v:imagedata src="file:///C:/Users/rcornman/AppData/Local/Temp/1/msohtmlclip1/01/clip_image001.png" o:title="Diagram Description automatically generated">

10.7717/peerj.17482/supp-2 Supplemental Information 2 Comparison of scaffold length and coverage patterns to the karyotype of *Lasiurus cinereus*. A) Scaffold length, relative coverage in a population data set, and coefficient of variation (CV) of coverage among the 23 individual *L. cinereus* samples in that data set, see text for details. The inferred chromosome type is as inferred by the author using the terminology of Bickham (1987) and based on a comparison between panel A and panel B. The rows are color-coded by chromosome type in each panel. B) Karyotype of *L. cinereus* with colored boxes added by the author to represent the distinct chromosome types postulated in panel A and discussed in Bickham (1987). Karyotype image reproduced by permission from Bickham (1987) (©: Oxford University Press). C) Variation in relative coverage of chromosomes in mapped reads from the population data set, with the accession for each individual sample listed on the horizontal axis. The lines highlight trends among samples and chromosomes, using four line colors that correspond to the chromosome types illustrated in the preceding panels. Thus, gray lines represent relative coverage for large metacentric chromosomes, blue lines represent coverage for medium metacentric chromosomes, green lines represent coverage for short acrocentric chromosomes, and the red line represent coverage for the X chromosome.

10.7717/peerj.17482/supp-3 Supplemental Information 3 *Trpc4* gene structures in bats and carnivores do not suggest annotation errors or divergence in exon number that could lead to paralogous codon alignments and thus invalid estimates of evolutionary rate. A. Screen captures of annotated *Trpc4* transcripts in representative carnivore and bat genomes showing similar gene architectures. Images are from the Genome Data Viewer webtool of the National Center for Biotechnology Information (NCBI). B. Screen capture of a TRPC4 protein isoform from cat aligned to a representative bat genome. Aligned segments of the protein query match only annotated exons in the subject genome.

10.7717/peerj.17482/supp-4 Supplemental Information 4 A strong shift in background nucleotide composition occurs in four of the thirteen bat taxa analyzed. A) Percentage of coding sequences that is G or C for analyzed genes. Genes are sorted by genomic order in *Lasiurus cinereus* (genes absent in this species are not shown). Species are colored by taxonomic clade as in Fig. 7. Phyllostomid species have higher GC content across the entire region, whereas higher GC content in Molossus is largely restricted to genes of the NF block (see text for details). B) GC content in representative primates and rodents, which show much less variation among taxa.

10.7717/peerj.17482/supp-5 Supplemental Information 5 The positive selection candidate *Amer3* is part of a block of eight genes that was rearranged in the ancestor of bats and has maintained tight linkage to the “CE” gene block through subsequent bat evolution. A. A table of the order (numbers) and orientation (plus or minus symbols) of eight landmark genes in outgroup tetrapods and in representative bat species. Green cells indicate the genes remain tightly linked to *Amer3* in that taxon, yellow cells indicate the genes are present in the genome but not linked to *Amer3*, and red cells indicate the genes were not found in that species. Dark green cells indicate the orientation of the gene differs from the presumed ancestral state. The black box around *Amer3* in *Lasiurus cinereus* denotes that the positive selection test was significant. B. A schematic of the relative positions of gene blocks on linkage groups of the genome assemblies of human and five representative bat species. Gene symbols for landmark genes based on human nomenclature define each block as shown in the legend. See text for details of how gene blocks are defined.

10.7717/peerj.17482/supp-6 Supplemental Information 6 Schematic of the organization of *Fgf9* and *Sacs* genes in bats and other tetrapods. Each ideogram displays a linear arrangement of landmark genes identified by gene symbol. Gene orientation is indicated by a plus or minus symbol, whereas numbers indicate the gene order in human to aid the visualization of rearrangements. Genes colored purple are those that remain tightly linked to *Fgf9* whereas genes colored blue remain tightly linked to *Sacs*. The first three genes of the CE (orange) and NF (green) blocks, as defined in the text, are shown when in proximity to the *Sacs* or *Fgf9* genes. Genes colored white are not consistently linked to any block but are useful for identifying additional structural rearrangements in the regions. Greyed genes were not found in a given species, whereas gray-colored gaps of the specified size in megabases (Mb) indicate a large span of intervening genomic sequence. Accession numbers for each linkage group are shown above each box, with multiple boxes indicating genes that are on different linkage groups. A. Representative tetrapod gene organizations. B Representative gene organizations in bats.

10.7717/peerj.17482/supp-7 Supplemental Information 7 Organization of conserved landmark genes in the vicinity of genes selected in *Lasiurus cinereus*, in bats and other tetrapod groups. Genes are organized into five color-coded multi-gene blocks, defined as described in the text and numbered according to their order in human. Arrowheads indicate relative orientation on plus or minus strands of each chromosome. Genes on the same linkage group are boxed within each species. Intergenic distances are not to scale and other genes that may be present in the region in a given species are not shown. Genes listed in the legend that are absent in any give taxon are either lost or located elsewhere in the genome, see text for details. A. Gene organization in nine bats. The twelve positive-selection candidates in *L. cinereus* have a darker outline. B. Organization of the same landmark genes in other tetrapods, indicating a much slower pace of structural evolution despite the greater evolutionary divergence time.

10.7717/peerj.17482/supp-8 Supplemental Information 8 Properties of Tbx1-like genes. A) Comparative secondary structure of the human TBX1 protein and the translation of an example Tbx1-like gene annotated by Cornman & Cryan (2022). Beta sheet motifs are represented by green arrows and alpha helix motifs are represented by red arrows. Unstructured amino-acid sequence at the C-terminus of each protein has been trimmed. Yellow boxes indicate residues predicted to interact with DNA targets (see text for details). The arginine residue (“R”) at position 137 is predicted to contact the major groove of the DNA helix. B) Alignments of a putative Tbx1-like homolog in *Pipistrellus kuhlii* to the Pipistrellus genome, as shown in the Genome Data Viewer of the National Center for Biotechnology Information. The scaffold and position of each match is shown. The bottom track is public RNA-Seq coverage data, showing RNA alignments overlapping the TBLASTN matches. C) A protein-level alignment of the coding sequence annotated in File S5 and the closest BLASTX matches in three other Vespertilionidae. Incomplete or frame-shifted codons are translated as “X”, whereas internal stop codons are translated as “*”. Residues using the same font color have similar biochemical properties, following the scheme used in BioEdit. Any use of trade, firm, or product names is for descriptive purposes only and does not imply endorsement by the U.S. Government.

10.7717/peerj.17482/supp-9 Supplemental Information 9 Sequence alignments and trees used in tests of diversifying selection. Sequence alignments are in FASTA format and guide trees are in newick format. Sequences are labeled with genus names only for consistency and software compatibility.

10.7717/peerj.17482/supp-10 Supplemental Information 10 Output and P-values for tests of diversifying selection described in the text. Program outputs are shaded in blue. *P*-values less than 0.01 are bolded. The first column indicates whether the gene was found to have significant evidence of diversifying selection in previous work, as discussed in the text. A separate sheet of output is given for each tree analyzed (see text for details). The final sheet gives the tree and PAML output for the Trpc4 gene comparison between bats and carnivores.

10.7717/peerj.17482/supp-11 Supplemental Information 11 Locations of genes and gene blocks discussed in the text for outgroup and bat species. Other bat species used in evolutionary rate analysis were not included in this synteny comparison if the orthologous genes were not located on large linkage groups. Note that Mab21l1 is nested within an intron of Nbea and thus are both labeled as position “1” in the column “Human order”. The strand and the order in human are listed to aid the detection of structural changes.

10.7717/peerj.17482/supp-12 Supplemental Information 12 Standard BLAST text output for searches of Tbx1-like sequence XP_027987819.1 against reference genomes of *Pipistrellus kuhlii* and *Myotis myotis*. The two outputs are concatenated here, each beginning with the line "Job Title".

10.7717/peerj.17482/supp-13 Supplemental Information 13 Annotation of standard gene features associated with a Tbx1-like sequence in the *Pipistrellus kuhlii* reference genome sequence. The predicted core promoter TATA box is highlighted in blue. The inferred transcription start site is underlined based on the standard -25 offset of the TATA box. The first transcribed start codon is highlighted in green. Two exons and a proposed intron are shown, the intron was inferred by alignment of the conceptual translation to other Tbx1-like predictions in *Lasiurus cinereus* and *Eptesicus fuscus*. A canonical polyadenylation signal is highlighted in yellow. See text for details.

## Additional Information and Declarations

### Data Availability

The following information was supplied regarding data availability: DNAZoo genome assembly of the hoary bat, available at https://www.dnazoo.org/assemblies/aeorestes_cinereus The short read data, PRJNA559902, were used to further evaluate the genome assembly.
