# Supplementary figures and images for: A genomic hotspot of diversifying selection and structural change in the hoary bat (Lasiurus cinereus)
Source: PeerJ. 2024 May 31;12:e17482. doi: 10.7717/peerj.17482 (PMC11146322; doi:10.7717/peerj.17482)

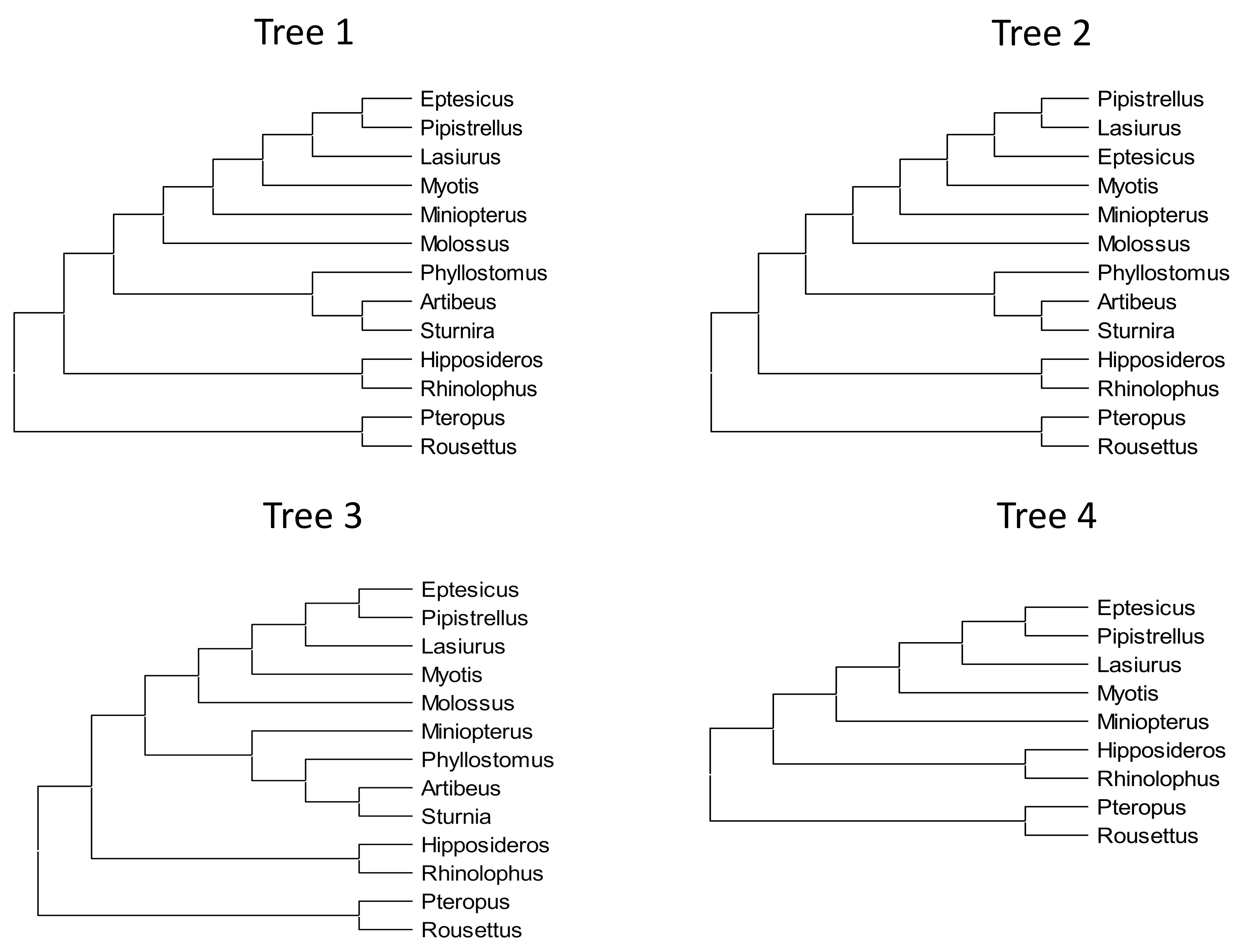

Supplement: Supplemental Information 1 — Tree 1 is consistent with Amador et al. (2018). Tree 2 differs from tree 1 only in postulating that Lasiurus is closer to Pipistrellus than the latter is to Eptesicus. Tree 3 is consistent with Agnarsson et al. (2011). Tree 4 is the same as Tree 1 except that four taxa with skewed nucleotide composition have been pruned (see text for details). [file peerj-12-17482-s001.png]

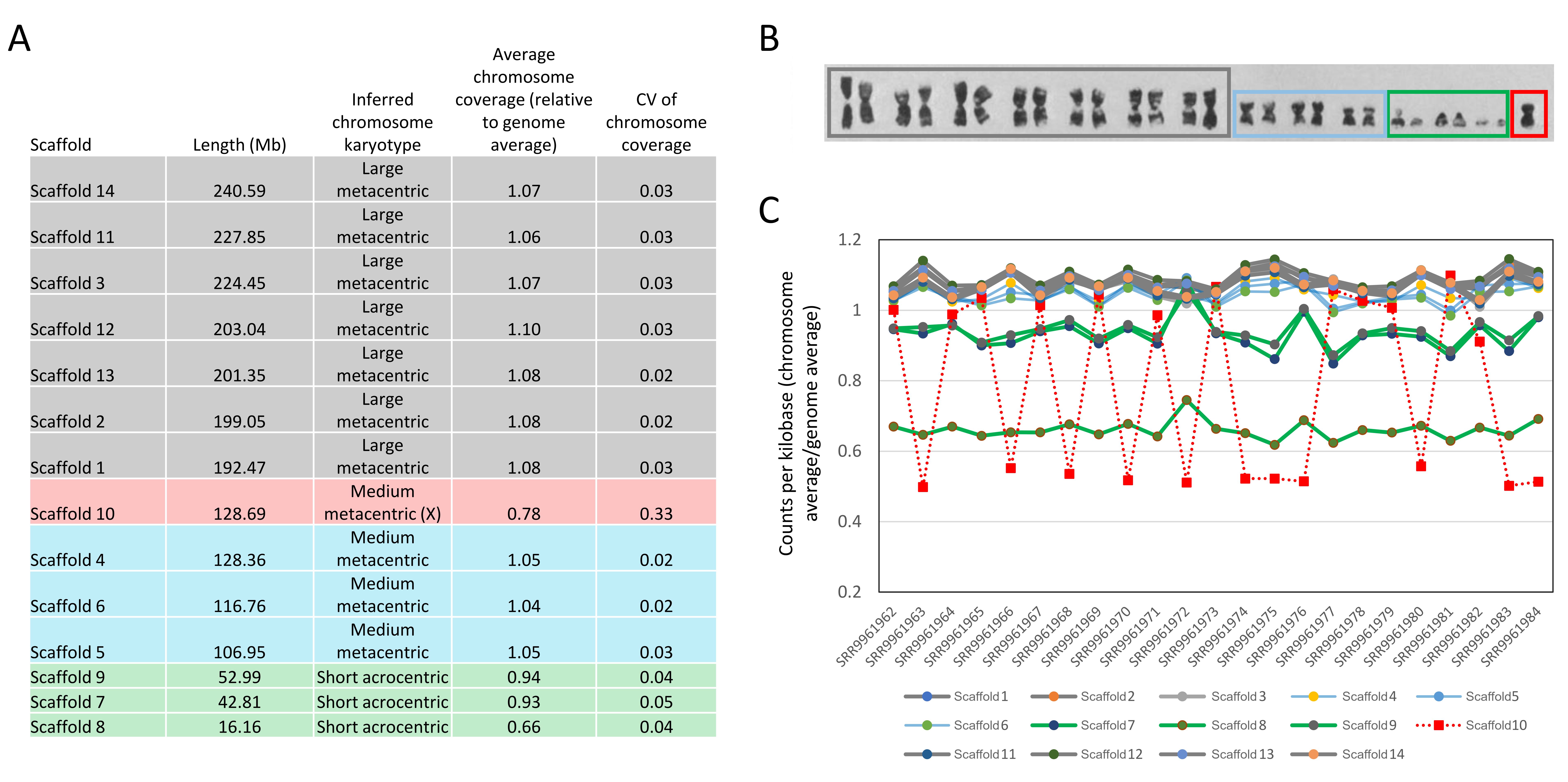

Supplement: Supplemental Information 2 — A) Scaffold length, relative coverage in a population data set, and coefficient of variation (CV) of coverage among the 23 individual L. cinereus samples in that data set, see text for details. The inferred chromosome type is as inferred by the author using the terminology of Bickham (1987) and based on a comparison between panel A and panel B. The rows are color-coded by chromosome type in each panel. B) Karyotype of L. cinereus with colored boxes added by the author to represent the distinct chromosome types postulated in panel A and discussed in Bickham (1987). Karyotype image reproduced by permission from Bickham (1987) (©: Oxford University Press). C) Variation in relative coverage of chromosomes in mapped reads from the population data set, with the accession for each individual sample listed on the horizontal axis. The lines highlight trends among samples and chromosomes, using four line colors that correspond to the chromosome types illustrated in the preceding panels. Thus, gray lines represent relative coverage for large metacentric chromosomes, blue lines represent coverage for medium metacentric chromosomes, green lines represent coverage for short acrocentric chromosomes, and the red line represent coverage for the X chromosome. [file peerj-12-17482-s002.png]

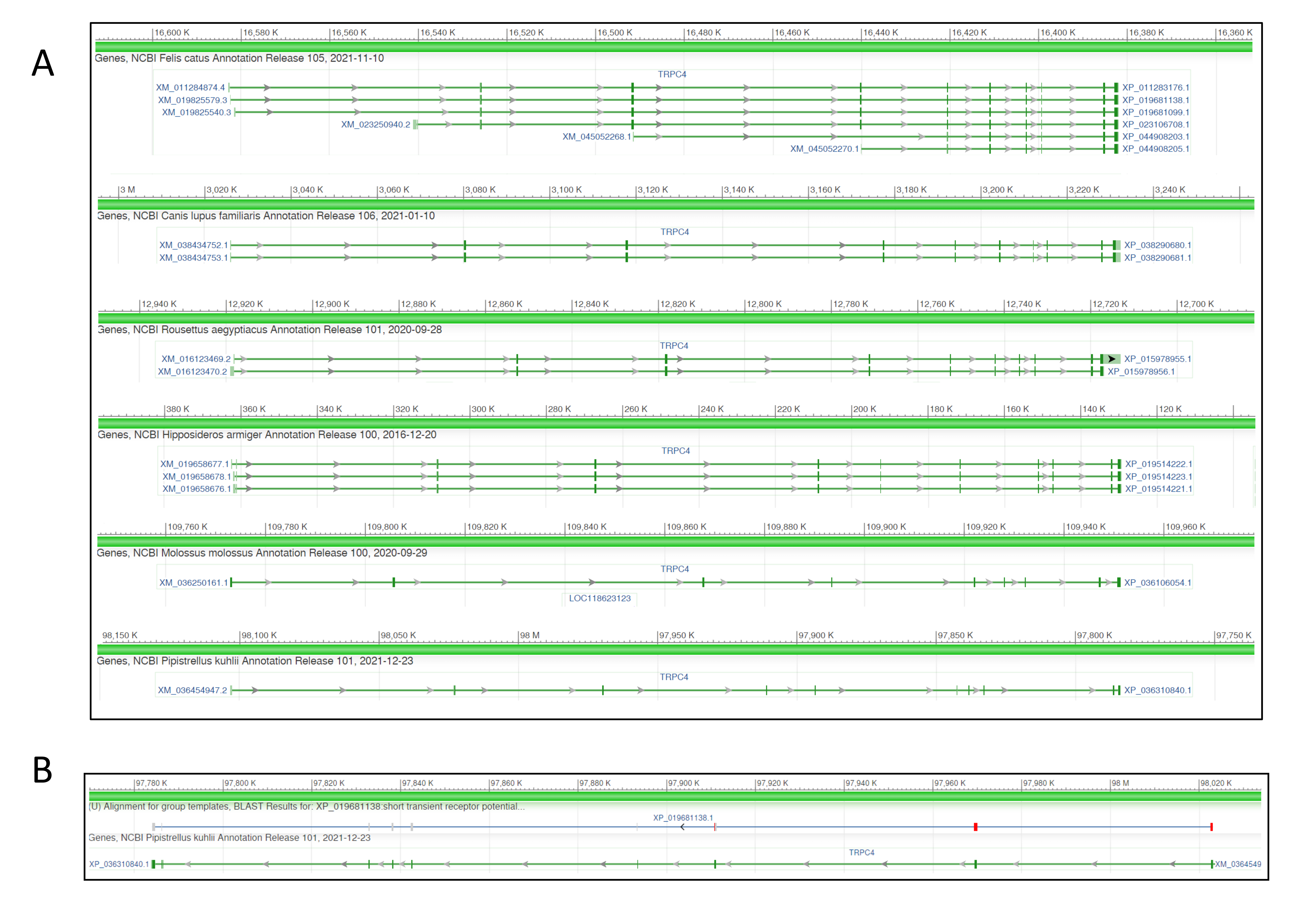

Supplement: Supplemental Information 3 — A. Screen captures of annotated Trpc4 transcripts in representative carnivore and bat genomes showing similar gene architectures. Images are from the Genome Data Viewer webtool of the National Center for Biotechnology Information (NCBI). B. Screen capture of a TRPC4 protein isoform from cat aligned to a representative bat genome. Aligned segments of the protein query match only annotated exons in the subject genome. [file peerj-12-17482-s003.png]

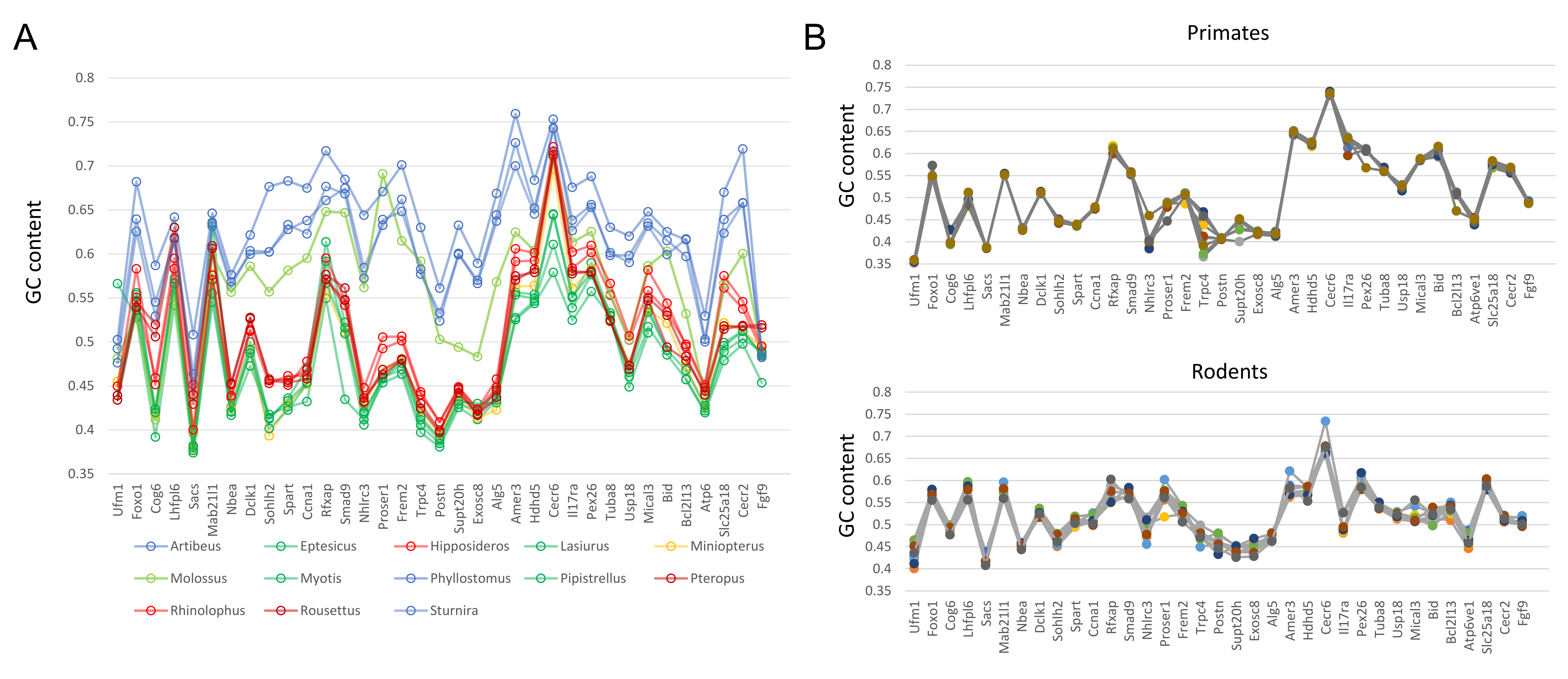

Supplement: Supplemental Information 4 — A) Percentage of coding sequences that is G or C for analyzed genes. Genes are sorted by genomic order in Lasiurus cinereus (genes absent in this species are not shown). Species are colored by taxonomic clade as in Fig. 7. Phyllostomid species have higher GC content across the entire region, whereas higher GC content in Molossus is largely restricted to genes of the NF block (see text for details). B) GC content in representative primates and rodents, which show much less variation among taxa. [file peerj-12-17482-s004.png]

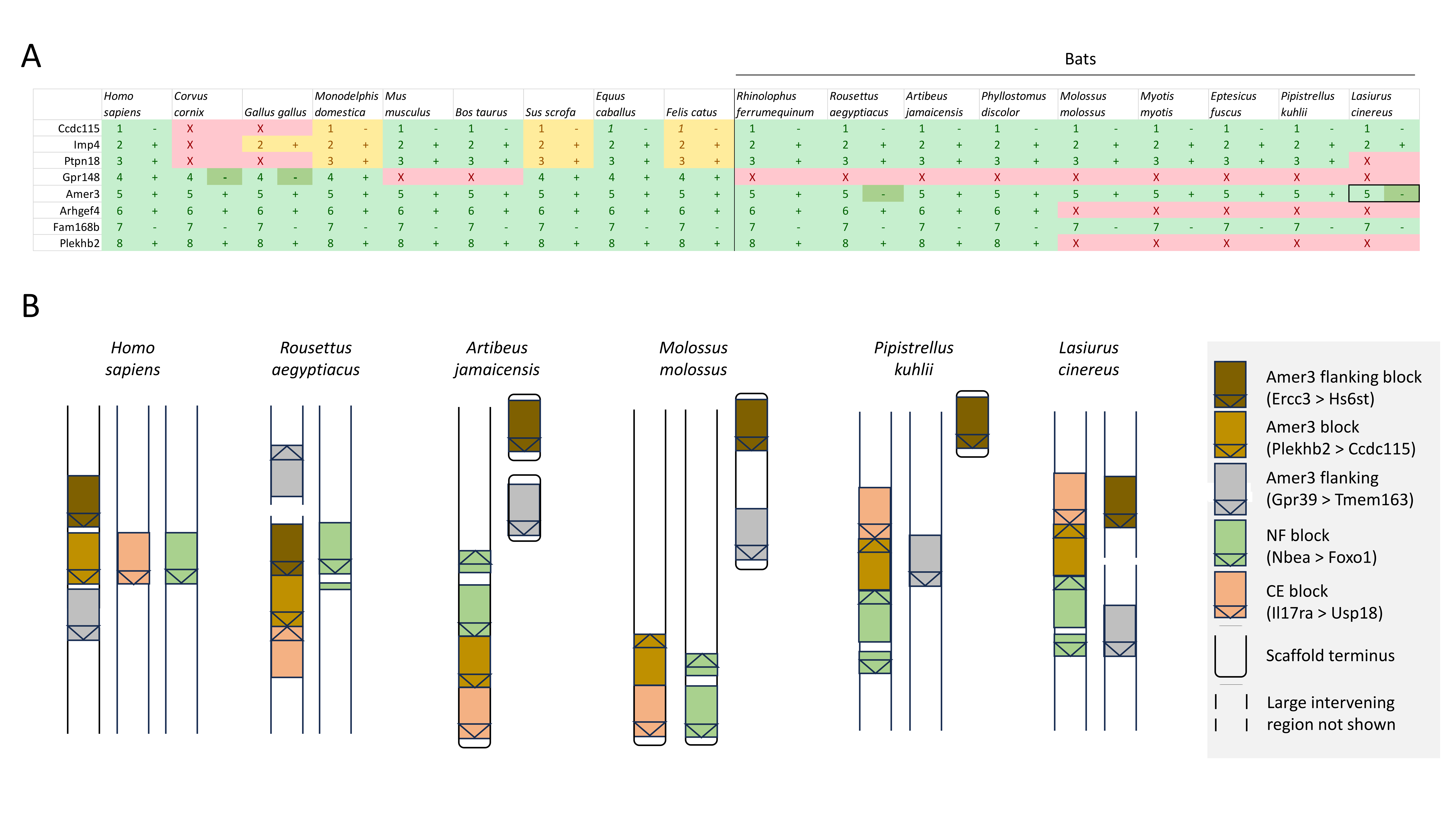

Supplement: Supplemental Information 5 — A. A table of the order (numbers) and orientation (plus or minus symbols) of eight landmark genes in outgroup tetrapods and in representative bat species. Green cells indicate the genes remain tightly linked to Amer3 in that taxon, yellow cells indicate the genes are present in the genome but not linked to Amer3, and red cells indicate the genes were not found in that species. Dark green cells indicate the orientation of the gene differs from the presumed ancestral state. The black box around Amer3 in Lasiurus cinereus denotes that the positive selection test was significant. B. A schematic of the relative positions of gene blocks on linkage groups of the genome assemblies of human and five representative bat species. Gene symbols for landmark genes based on human nomenclature define each block as shown in the legend. See text for details of how gene blocks are defined. [file peerj-12-17482-s005.png]

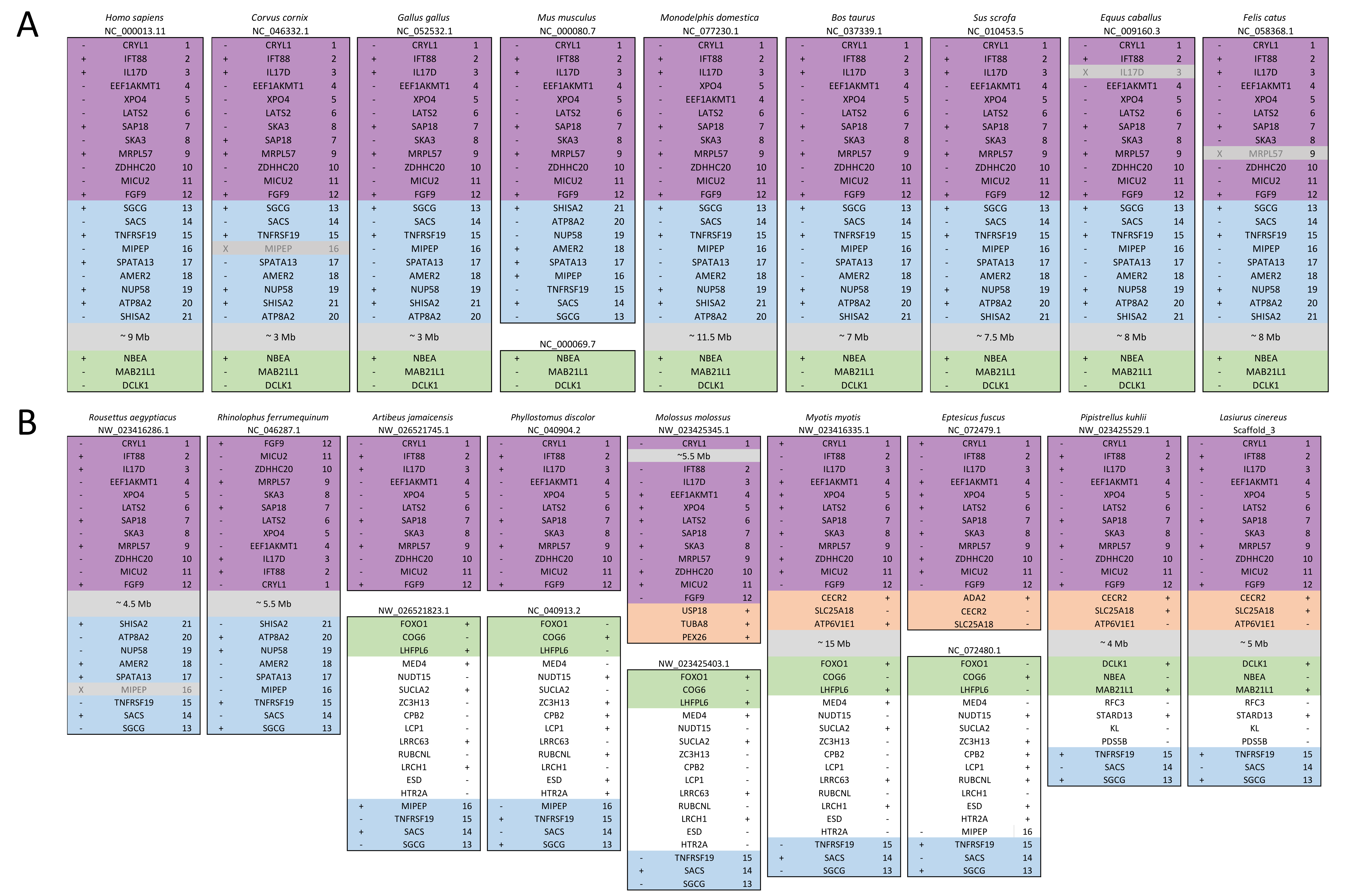

Supplement: Supplemental Information 6 — Each ideogram displays a linear arrangement of landmark genes identified by gene symbol. Gene orientation is indicated by a plus or minus symbol, whereas numbers indicate the gene order in human to aid the visualization of rearrangements. Genes colored purple are those that remain tightly linked to Fgf9 whereas genes colored blue remain tightly linked to Sacs. The first three genes of the CE (orange) and NF (green) blocks, as defined in the text, are shown when in proximity to the Sacs or Fgf9 genes. Genes colored white are not consistently linked to any block but are useful for identifying additional structural rearrangements in the regions. Greyed genes were not found in a given species, whereas gray-colored gaps of the specified size in megabases (Mb) indicate a large span of intervening genomic sequence. Accession numbers for each linkage group are shown above each box, with multiple boxes indicating genes that are on different linkage groups. A. Representative tetrapod gene organizations. B Representative gene organizations in bats. [file peerj-12-17482-s006.png]

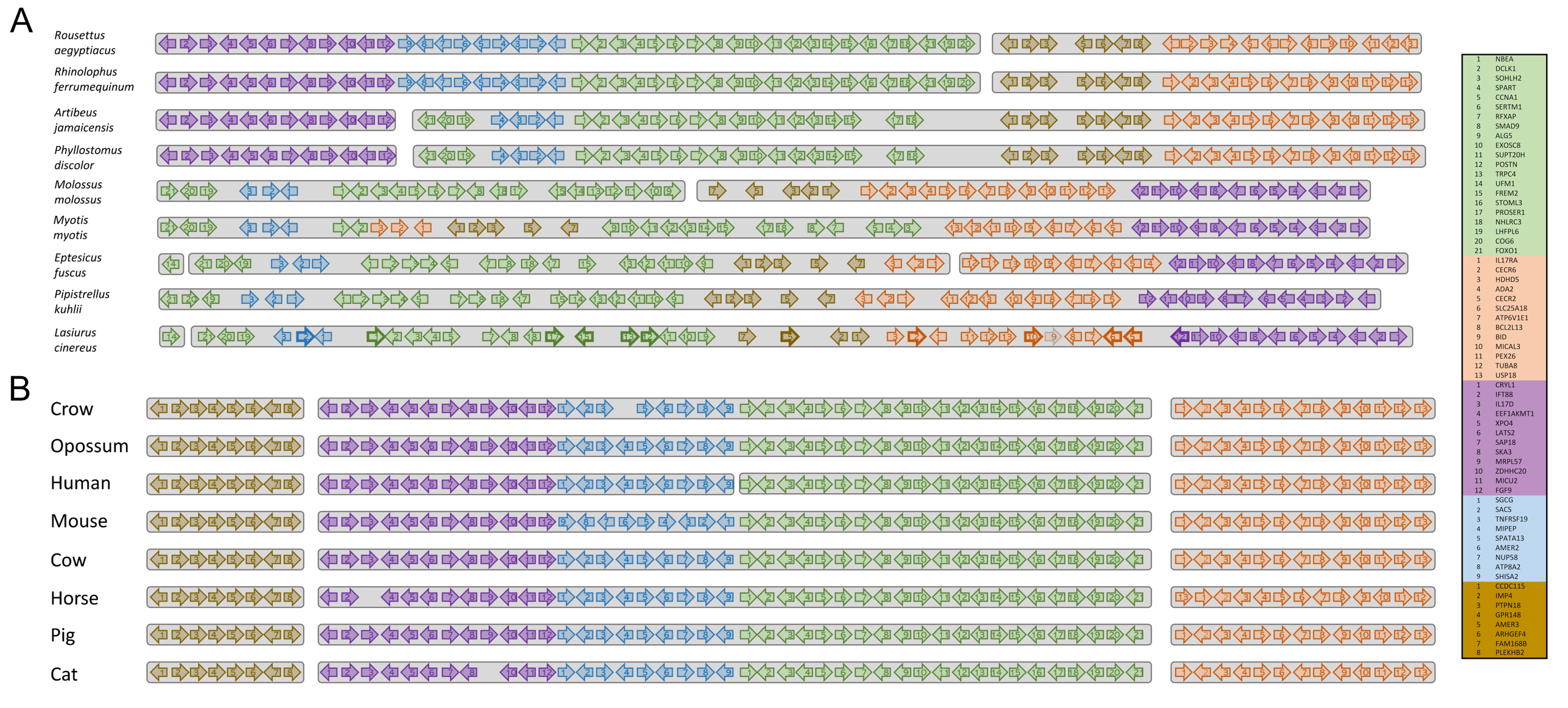

Supplement: Supplemental Information 7 — Genes are organized into five color-coded multi-gene blocks, defined as described in the text and numbered according to their order in human. Arrowheads indicate relative orientation on plus or minus strands of each chromosome. Genes on the same linkage group are boxed within each species. Intergenic distances are not to scale and other genes that may be present in the region in a given species are not shown. Genes listed in the legend that are absent in any give taxon are either lost or located elsewhere in the genome, see text for details. A. Gene organization in nine bats. The twelve positive-selection candidates in L. cinereus have a darker outline. B. Organization of the same landmark genes in other tetrapods, indicating a much slower pace of structural evolution despite the greater evolutionary divergence time. [file peerj-12-17482-s007.png]

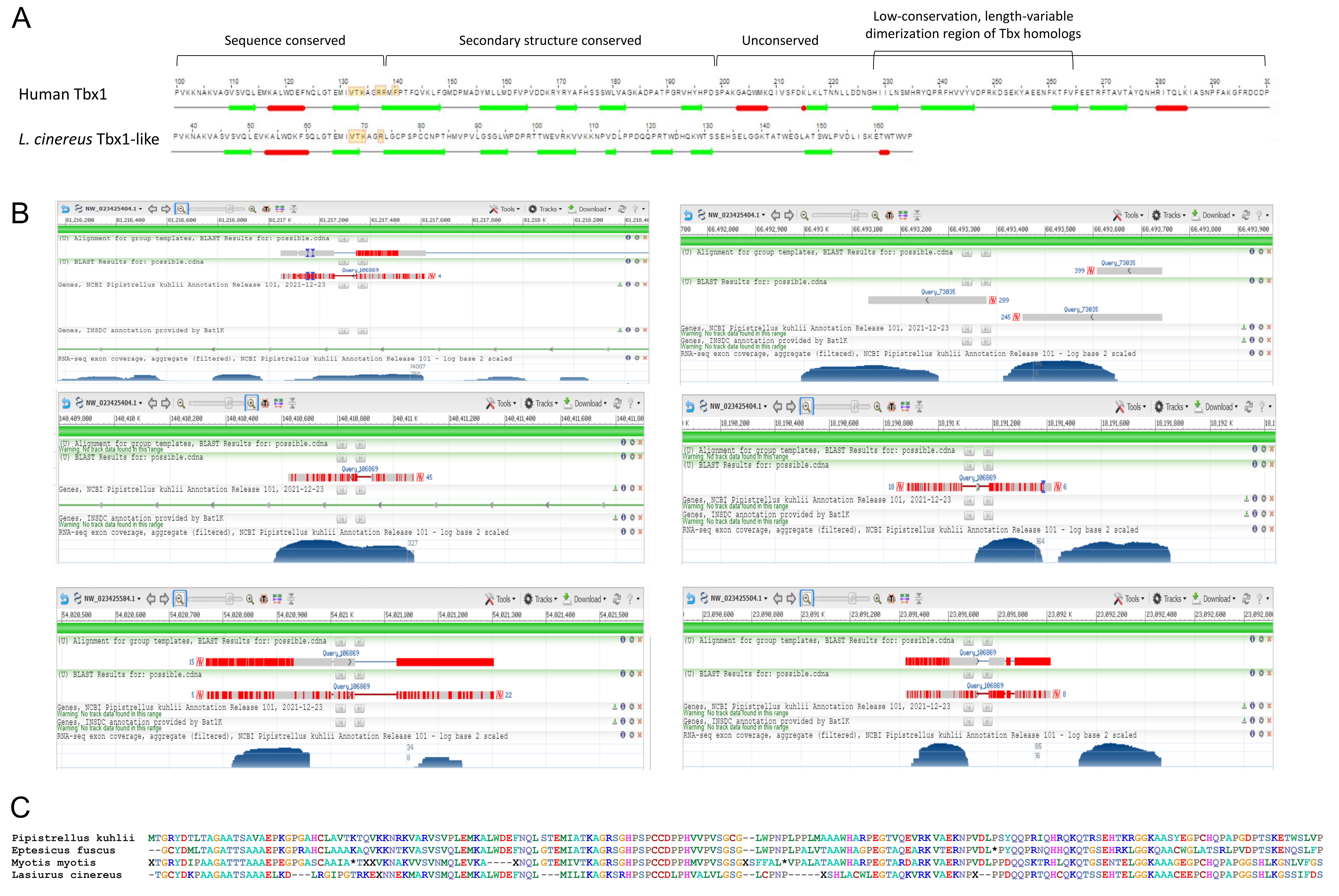

Supplement: Supplemental Information 8 — A) Comparative secondary structure of the human TBX1 protein and the translation of an example Tbx1-like gene annotated by Cornman & Cryan (2022). Beta sheet motifs are represented by green arrows and alpha helix motifs are represented by red arrows. Unstructured amino-acid sequence at the C-terminus of each protein has been trimmed. Yellow boxes indicate residues predicted to interact with DNA targets (see text for details). The arginine residue (“R”) at position 137 is predicted to contact the major groove of the DNA helix. B) Alignments of a putative Tbx1-like homolog in Pipistrellus kuhlii to the Pipistrellus genome, as shown in the Genome Data Viewer of the National Center for Biotechnology Information. The scaffold and position of each match is shown. The bottom track is public RNA-Seq coverage data, showing RNA alignments overlapping the TBLASTN matches. C) A protein-level alignment of the coding sequence annotated in File S5 and the closest BLASTX matches in three other Vespertilionidae. Incomplete or frame-shifted codons are translated as “X”, whereas internal stop codons are translated as “*”. Residues using the same font color have similar biochemical properties, following the scheme used in BioEdit. Any use of trade, firm, or product names is for descriptive purposes only and does not imply endorsement by the U.S. Government. [file peerj-12-17482-s008.png]
