# Supplementary material for: A genomic hotspot of diversifying selection and structural change in the hoary bat (Lasiurus cinereus)
Source: PeerJ. 2024 May 31;12:e17482. doi: 10.7717/peerj.17482 (PMC11146322; doi:10.7717/peerj.17482)
Supplement: Supplemental Information 13 — The predicted core promoter TATA box is highlighted in blue. The inferred transcription start site is underlined based on the standard -25 offset of the TATA box. The first transcribed start codon is highlighted in green. Two exons and a proposed intron are shown, the intron was inferred by alignment of the conceptual translation to other Tbx1-like predictions in Lasiurus cinereus and Eptesicus fuscus. A canonical polyadenylation signal is highlighted in yellow. See text for details. [file peerj-12-17482-s013.docx]

Supplemental File S5. Annotation of standard gene features associated with a Tbx1-like sequence in the *Pipistrellus kuhlii* reference genome sequence. The predicted core promoter TATA box is highlighted in blue. The inferred transcription start site is underlined based on the standard -25 offset of the TATA box. The first transcribed start codon is highlighted in green. Two exons and a proposed intron are shown, the intron was inferred by alignment of the conceptual translation to other Tbx1-like predictions in *Lasiurus cinereus* and *Eptesicus fuscus*. A canonical polyadenylation signal is highlighted in yellow. See text for details.

Source: NW_023425404.1: 66492177- 66493798 (complement), isolate mPipKuh1 unplaced genomic scaffold, mPipKuh1.p scaffold_m20_p_1, whole genome shotgun sequence

Core promoter:

TTTGTGTTCTGGGCCTCTTGTC TATAGAATAAAA GCCCAGTGACTGTC

Exon1:

ACGGCAGAATGCTGGTCGCTATGACACGCTGACTGCAGGGGCCGCCACCAGTGCTGTTGCTGAGCCCAAGGGCCCTGGGGCCCATTGCCTGGCTGTCACCAAGACACAGGTAAAGAAGAACAGAAAGGTGGCCAGGGTGAGCGTGCCGCTTGAGATGAAGGCTCTGTGGGATGAATTCAATCAGCTGAGCACCGAGATGATCGCCACCAAGGCTGGCAGGTCAGGGCACCCCTCTCCCTGCTGTGACCCTCCCCATGTGGTACCAGTCTCAGGCTGCGGGCTTTGGCCCAATCCAC

Possible intron:

GTCAGCAAGACTAAGAAGTGGGGCCGGGGGGACACAGCCTGGCCACTTGCAGGTCCAGTTTCTTCACTCTCTGAG

Exon2:

TCCCACCTCTAATGGCAGCCGCCTGGCATGCCAGACCAGAGGGTACTGTTCAGGAGGTCAGGAAGGTGGCAGAGAAAAACCCTGTGGATCTGCCATCCTACCAGCAGCCCAGGATTCAGCACCGCCAGAAACAGACTCGCTCAGAGCACACCAAGCGTGGGGGGAAGGCAGCCTCCTATGAGGGGCCTTGCCACCAGCCAGCTCCGGGGGATCCCACCTCAAAGGAAACTTGGTCTTTGGTTCCTTAGTTCAGTCTATCTCAGCTAAAGAGGAGCAGCTGAGCACATTTTAGCGGAGAGTTAAGGCTCTAAAGCAGTGTGTTCTGTTCTGTGGAGATTGCTACCCAGTTGTGTTAGGCCTAAATTCCAGGTGATTGTGGGGCCTGGCTGGCTCTGCCACTGGAACCCACATCTTCCACCTGGCCTGTATTTATTGTAAGGGCACGAAGAGAAGGGGAGCAAGTTTTTTGATGCAATGTATTTGACCAACAGACAAAGGTGAGTTCCAGACTGTGTCTAATATAGTCACAGAGACACCAGCTCTGAGTCTGCCTGGCCAAAGGGAGCTCAGAGCCTGTTTGCAGAGCCTTCTGGTGGTTTCTCTTCCCTTTCTGAGGCCATCCTCCCTGGTAAGTCTGGGGCCCATTCTCATATCTAGATCATTGGGTTTTCTTCAGGACCCTCCCCCTAGCTGGCCCTGTTCCTGATAGGCCCCCAGCCCCACCCCGATCTGGGATGGGACTGGCTGGCCAACTTCCAGTGGCCCATCCCCCCTGCCCTGCCCAGCCCAATTGCCTCAATCAGTGCAAGGCTGCCAGACCCCACCCATACACAAATTCACGCACCAGGCCTCTAGTTTAATATAATGAGCTTAGCTATTTCGACCCCATTCTGTTTTACTTATATATCTCATGCTTAATAGAACCTTTTCAAGTACACTTAGTTTAAAATTGGATTCTGATACCTAAATGCTGAGACTGAGCTCTTTAGGAGAATCCTTATTCTAAATGAATTTAGTGATAAATAGAGGTCCTGCCTCTCTAATCCAACACAGATTTTTAGCATGCACCTTTGTGAATCTGCCCAGTGGTATAGCTGTACAAATGCTATTCTTGCTGAATAATCTAGAGTTTGAGTGTCTGTAGAATATCCACTGGGAATTGTTCCAAGACCTAGATCTAAAATATTATACACCAAAATAAA

Proposed cDNA:

ATGACTGGTCGCTATGACACGCTGACTGCAGGGGCCGCCACCAGTGCTGTTGCTGAGCCCAAGGGCCCTGGGGCCCATTGCCTGGCTGTCACCAAGACACAGGTAAAGAAGAACAGAAAGGTGGCCAGGGTGAGCGTGCCGCTTGAGATGAAGGCTCTGTGGGATGAATTCAATCAGCTGAGCACCGAGATGATCGCCACCAAGGCTGGCAGGTCAGGGCACCCCTCTCCCTGCTGTGACCCTCCCCATGTGGTACCAGTCTCAGGCTGCGGGCTTTGGCCCAATCCACTCCCACCTCTAATGGCAGCCGCCTGGCATGCCAGACCAGAGGGTACTGTTCAGGAGGTCAGGAAGGTGGCAGAGAAAAACCCTGTGGATCTGCCATCCTACCAGCAGCCCAGGATTCAGCACCGCCAGAAACAGACTCGCTCAGAGCACACCAAGCGTGGGGGGAAGGCAGCCTCCTATGAGGGGCCTTGCCACCAGCCAGCTCCGGGGGATCCCACCTCAAAGGAAACTTGGTCTTTGGTTCCT

Translation:

MTGRYDTLTAGAATSAVAEPKGPGAHCLAVTKTQVKKNRKVARVSVPLEMKALWDEFNQLSTEMIATKAGRSGHPSPCCDPPHVVPVSGCGLWPNPLPPLMAAAWHARPEGTVQEVRKVAEKNPVDLPSYQQPRIQHRQKQTRSEHTKRGGKAASYEGPCHQPAPGDPTSKETWSLVP
